# Synthesis and Biological Activity of New Hydrazones Based on N-Aminomorpholine

**DOI:** 10.3390/molecules29153606

**Published:** 2024-07-30

**Authors:** Oralgazy A. Nurkenov, Saule B. Zhautikova, Andrei I. Khlebnikov, Ardak K. Syzdykov, Serik D. Fazylov, Tulegen M. Seilkhanov, Saule K. Kabieva, Kobylandy M. Turdybekov, Anel Z. Mendibayeva, Gaziza M. Zhumanazarova

**Affiliations:** 1 Institute of Organic Synthesis and Coal Chemistry of the Republic of Kazakhstan, Karaganda 100008, Kazakhstan; nurkenov_oral@mail.ru (O.A.N.); ardak.syzdykov.96@inbox.ru (A.K.S.); iosu8990@mail.ru (S.D.F.); anenyawa@mail.ru (A.Z.M.); 2 Karaganda Industrial University, Temirtau 101400, Kazakhstan; kabieva.s@mail.ru; 3 Karaganda Medical University, Karaganda 100024, Kazakhstan; 4 Kizhner Research Center, National Research Tomsk Polytechnic University, Tomsk 634050, Russia; aikhl@chem.org.ru; 5 Faculty of Chemistry, National Research Tomsk State University, Tomsk 634050, Russia; 6 Sh. Ualikhanov Kokshetau University, Kokshetau 020000, Kazakhstan; tseilkhanov@mail.ru; 7 Karaganda Buketov University, Karaganda 100028, Kazakhstan; xray-phyto@yandex.kz

**Keywords:** N-aminomorpholine, hydrazones, functionally substituted benzaldehydes, o-formyl benzoic acid, 3-acetoxy-isoindolin-1-ones, NMR spectroscopy, antiviral activity, molecular modeling

## Abstract

The data on the synthesis of N-aminomorpholine hydrazones are presented. It is shown that the interaction of N-aminomorpholine with functionally substituted benzaldehydes and 4-pyridinaldehyde in isopropyl alcohol leads to the formation of corresponding hydrazones. The structure of the synthesized compounds was studied by ^1^H and ^13^C NMR spectroscopy methods, including the COSY (^1^H-^1^H), HMQC (^1^H-^13^C) and HMBC (^1^H-^13^C) methodologies. The values of chemical shifts, multiplicity, and integral intensity of ^1^H and ^13^C signals in one-dimensional NMR spectra were determined. The COSY (^1^H-^1^H), HMQC (^1^H-^13^C), and HMBC (^1^H-^13^C) results revealed homo- and heteronuclear interactions, confirming the structure of the studied compounds. The antiviral, cytotoxic, and antimicrobial activity of some synthesized hydrazones were investigated. It is shown that 2-((morpholinoimino)methyl)benzoic acid has a pronounced viral inhibitory property, comparable in its activity to commercial drugs Tamiflu and Remantadine. A docking study was performed using the influenza virus protein models (1930 Swine H1 Hemagglutinin and Neuraminidase of 1918 H1N1 strain). The potential binding sites that are complementary with 2-((morpholinoimino)methyl)benzoic acid were found.

## 1. Introduction

One of the crucial objectives in contemporary organic and medicinal chemistry is the challenge of creating novel biologically active compounds to design targeted medications for addressing prevalent diseases in society. 

One of the most promising approaches to develop new pharmacologically significant agents consists in using available heterocyclic compounds as starting substances [1].

The use of heterocyclic compounds as starting materials in drug discovery and development is a common and successful strategy in medicinal chemistry. Heterocyclic compounds are versatile building blocks that can be modified to create diverse chemical structures with a wide range of biological activities. By making subtle changes to the structure of a heterocyclic compound, researchers can fine-tune its properties to optimize its pharmacological effects.

Heterocyclic compounds are prevalent in nature and are found in many biologically active molecules, making them attractive starting points for drug design. By exploring the chemical space around these compounds, researchers can identify new drug candidates with improved potency, selectivity, and safety profiles [2,3,4].

Overall, the use of heterocyclic compounds in drug discovery offers a powerful approach to developing new pharmacologically significant agents with the potential to treat various diseases effectively.

These substances can include morpholine, and its derivatives are a class of heterocyclic compounds that have shown diverse pharmacological effects and therapeutic potential. Their broad spectrum of activities makes them attractive candidates for further research and drug development in various therapeutic areas.

Morpholine derivatives have demonstrated effectiveness as nootropics, which are substances that can enhance cognitive function, memory, creativity, or motivation. Additionally, these compounds have shown promise in treating a range of diseases affecting different organ systems, including the respiratory system, gastrointestinal tract, and cardiovascular system, inflammatory conditions, immune disorders, and diseases of the central nervous system.

The versatility and pharmacological properties of morpholine derivatives make them valuable targets for medicinal chemistry research. Further exploration of these compounds could lead to the development of new drugs with improved efficacy and safety profiles for the treatment of a wide range of medical conditions [5]. Hydrazones derived from isonicotinic acid hydrazide (isoniazid) and nicotinic acid hydrazide have shown significant pharmacological activities and are utilized in the development of various drugs [6,7,8,9]. Here is a brief overview of their applications in different therapeutic areas: anti-tuberculosis activity, anti-inflammatory properties, anticonvulsant activity, and antidepressant effects [10,11,12,13,14]. In this regard, in continuation of the previously initiated studies [15,16,17], we have synthesized new biologically active hydrazones of N-aminomorpholine (**8**–**13**).

## 2. Results and Discussion

The condensation reaction of N-aminomorpholine (**1**) with functionally substituted benzaldehydes **2**–**5**, **7**, and 4-pyridinaldehyde (**6**) was carried out by heating equimolar amounts of the starting compounds in isopropyl alcohol at 60–70 °C for 3–5 h. The synthesis of new N-aminomorpholine hydrazones is shown in Table 1.

The composition and structure of compounds **8**–**13** were confirmed using the IR, NMR ^1^H, and NMR ^13^C methods, as well as by the data of COSY (^1^H-^1^H), HMQC (^1^H-^13^C), and HMBC (^1^H-^13^C).

**Figure 1:**
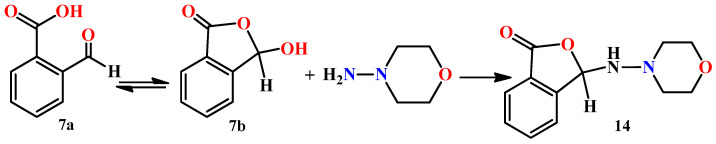
One of the possible directions of the interaction between N-aminomorpholine (**1**) and o-formyl benzoic acid.

Interesting results were obtained on the reaction of N-aminomorpholine (**1**) with o-formyl benzoic acid (**7**). In this case, the interaction is possible with the formation of compound **14**, since o-formyl benzoic acid (**7a**) forms aminophthalides in the reactions with some primary aromatic amines (Figure 1) [18].

For example, the interaction of **7a** with 2-aminothiophene derivatives gives an aminophthalide structure [19]. On the other hand, described the reaction of 2-aminopyrroles with compound **7a**, where Schiff bases were obtained as the major products, i.e., the o-formyl benzoic acid reacted in its open form. In references [20] it was shown that, unlike colorless aminophthalides, all of the products had a bright yellow color and characteristic NMR signals of the azomethine protons at about 9 ppm. The Schiff bases of o-formyl benzoic acid with p-phenylenediamine derivatives were also described in the literature [21]. In the reaction with anthranilic acid hydrazide, o-formyl benzoic acid **7** forms a normal hydrazone, i.e., a derivative of the open tautomeric form **7a**, which is described in the literature [22]. Therefore, when N-aminomorpholine (**1**) interacts with the open form of o-formyl benzoic acid **7a**, the formation of hydrazone **13** is expected.

To confirm the structure of hydrazone **13**, we applied different variants of the NMR method. Thus, in the ^1^H NMR spectrum of compound **13**, there were two doublets of protons of the morpholine fragment H^3,3,5,5^ and H^2,2,6,6^ at 3.05 and 3.72 ppm, respectively, with ^3^J 3.4 Hz. The signals of aromatic protons were observed as multiplets at 7.29–7.34 (H^11^), 7.44–7.94 (H^12^), 7.77–7.80 (H^10^), and 7.85–7.88 (H^13^) ppm. The azomethine proton H^8^ gives a multiplet at 8.31–8.32 ppm. The integral intensities of the mentioned signals correspond to the structure of compound **13**. The carboxylic proton H^17^ did not appear in the spectrum due to deuterium exchange or a salt formation.

In the ^13^C NMR spectrum of compound **13**, the signals of morpholine carbon atoms appeared at 51.93 (C^3,5^) and 66.16 (C^2,6^) ppm. Aromatic carbon atoms gave signals at 126.22 (C^13^), 128.10 (C^11^), 130.78 (C^10^), 132.13 (C^12,14^), and 134.70 (C^9^) ppm. The signal of the C^8^ azomethine atom was observed at 136.72 ppm. In the low-field region, the signal of the carboxylic atom C^15^ was detected at 168.99 ppm.

**Figure 2:**
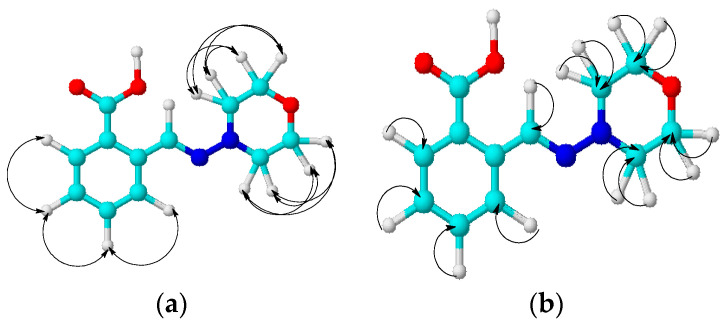
The correlation scheme in the COSY (**a**) and HMQC (**b**) spectra of compound **13**.

The structure of compound **13** was also confirmed by the methodologies of two-dimensional NMR, COSY (^1^H-^1^H) and HMQC (^1^H-^13^C), which allow establishing spin–spin interactions of a homo- and heteronuclear nature. Some of the observed correlations in the molecule are shown in Figure 2.

In the ^1^H-^1^H COSY spectra of compound **13**, we observed the proton spin–spin correlations through three bonds of neighboring methylene-methylene morpholine and methine-methine aromatic groups H^3,5^-H^2,6^ (3.05, 3.72 and 3.72, 3.05), H^11^-H^12^ (7.30, 7.46 and 7.46, 7.30), H^11^-H^10^ (7.31, 7.77 and 7.77, 7.31), and H^12^-H^13^ (7.46, 7.85 and 7.85, 7.46) ppm. Heteronuclear interactions of protons with carbon atoms through a single bond were found in ^1^H-^13^C HMQC spectra for the following pairs of nuclei: H^3,5^-C^3,5^ (3.03, 52.50), H^2,6^-C^2,6^ (3.71, 66.36), H^11^-C^11^ (7.30, 128.46), H^12^-C^12^ (4.46, 132.56), H^10^-C^10^ (7.76, 131.27), H^13^-C^13^ (7.85, 126.53), and H^8^-C^8^ (8.29, 135.30) ppm.

**Figure 3:**
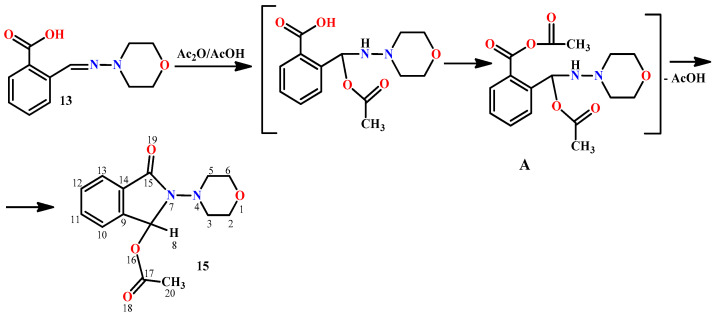
The cyclization of hydrazone **13** into phthalimidine **15**.

Next, we studied the interaction of hydrazone **13** with acetic anhydride, which leads to phthalimidine **15**. It was shown that the reaction proceeds smoothly only in the presence of a certain amount of acetic acid in the anhydride. With freshly distilled anhydride, compound **13** interacts with difficulty. The role of acetic acid, possibly, consists of the addition to the Schiff base. The resulting hydrazine can further form a mixed anhydride **A**, which undergoes cyclization into the final phthalimidine **15** with the elimination of acetic acid (Figure 3).

The ^1^H NMR spectrum of compound **15** contains a singlet at 2.10 ppm assigned to the acetoxy group protons H^20,20,20^. The signals of H^3ax,5ax^ and H^3eq,5eq^ in the morpholine fragment were observed as two multiplets at 3.18–3.23 and 3.29–3.35 ppm, respectively. The remaining morpholine protons H^2ax,6xa,2eq,6eq^ gave a multiplet at 3.58–3.62 ppm. The singlet at 7.01 ppm was assigned to the tertiary hydrogen atom H^8^. The multiplets of aromatic protons were observed at 7.50–7.51 (H^13^), 7.55–7.58 (H^11^), 7.62–7.64 (H^12^), and 7.65–7.67 (N^10^) ppm. The integral intensities of the signals are in agreement with the structural formula of compound **15**.

In the ^13^C NMR spectrum of compound **15**, the signals of morpholine carbon atoms appeared at 52.43 (C^3.5^) and 67.13 (C^2.6^) ppm. Carbon atoms of the acetoxy group gave signals at 21.34 (C^20^) and 170.95 (C^17^) ppm, while the signal of the tertiary carbon atom C^8^ appeared at 81.22 ppm. Carbon atoms of the aromatic ring were detected at 123.6 (C^12^), 124.49 (C^13^), 130.92 (C^11^), 131.31 (C^14^), 133.57 (C^10^), and 140.26 (C^9^) ppm. In the low-field region, the signal of the amide atom C^15^ was observed at 166.18 ppm.

**Figure 4:**
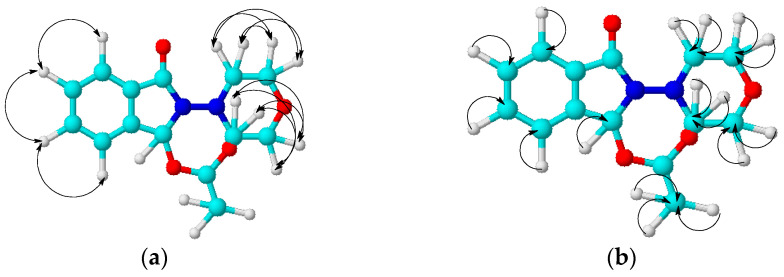
The correlation scheme in the COSY (**a**) and HMQC (**b**) spectra of compounds **15**.

The structure of compound **15** was also confirmed by the two-dimensional NMR methodologies COSY (^1^H-^1^H), HMQC (^1^H-^13^C), and HMBC (^1^H-^13^C). Some of the observed correlations in the molecule are shown in Figure 4.

In the ^1^H-^1^H COSY spectra of compound **15**, the spin–spin correlations of protons through three bonds of neighboring methylene-methylene morpholine and methine-methine aromatic groups were observed: H^3ax,5ax-^H^3eq,5eq^ (3.20, 3.31 and 3.31, 3.20), H^3ax,5ax-^H^2ax,6ax^ (3.20, 3.59 and 3.59, 3.20), H^3eq,5eq-^H^2eq,6eq^ (3.30, 3.60 and 3.60, 3.30), H^13^-H^12^ (7.49, 7.62 and 7.62, 7.49), and H^11^-H^10^ (7.57, 7.66 and 7.66, 7.57) ppm. Heteronuclear H^1^-C^13^ interactions through a single bond were established using ^1^H-^13^C HMQC spectroscopy for the following atom pairs: H^20^-C^20^ (2.09, 21.58), H^3ax,5ax-^C^3,5^ (3.19, 52.28), H^3eq,5eq-^C^3,5^ (3.31, 52.41), H^2ax,2eq,6ax,6eq^-C^2,6^ (3.58, 67.16), H^11^-C^11^ (7.56, 130.94), H^12^-C^12^ (7.65, 13.32), H^10^-C^10^ (7.63, 133.56), H^13^-C^13^ (7.50, 124.48), and H^8^-C^8^ (7.00, 81.47) ppm. Heteronuclear interactions of protons with carbon atoms through two or more bonds were detected using ^1^H-^13^C HMBC spectroscopy for the following pairs present in the compound: H^20^-C^17^ (2.09, 171.83), H^8^-C^14^ (6.99, 131.77), H^8^-C^9^ (6.99, 140.75), H^8^-C^15^ (7.00, 166.73), and H^8^-C^17^ (7.00, 171.35) ppm.

**Figure 5:**
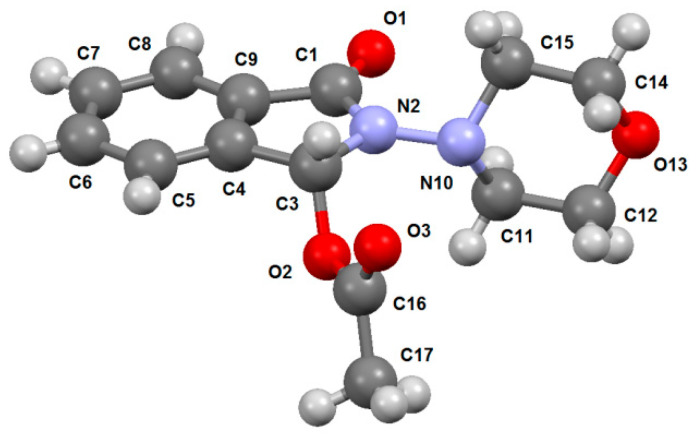
The spatial structure of molecule **15** determined by means of X-ray diffraction analysis (the ellipsoids of thermal vibrations are shown with a probability of 30%).

In order to determine the spatial structure of 2-morpholino-3-oxoisoindoline-1-yl acetate **15**, its single crystal X-ray diffraction study was carried out (Figure 5).

The X-ray data indicate that the bond lengths and valence angles in molecule **15** are close to the typical values as described in literature [23]. 

The conformation of the morpholine cycle in compound **15** is close to the ideal chair (ΔC_S_^10^ = 0.6° (max) and ΔC_2_^10,15^ = 0.8° (max)). The phthalimidine moiety adopts an equatorial orientation relative to the morpholine cycle. The phthalimidine fragment, except the sp^3^ hybridized C^3^ atom of the five-membered ring, remains planar. The deviations of the atoms from the middle plane are within ±0.014 Å. The corresponding value in the crystal structure of phthalimide is ±0.007 Å as stated in the literature [24]. The configuration of the N^2^ atom in molecule **15** is trigonal (the sum of the valence angles is 360.0°).

Heterocyclic compounds are of great importance for pharmacology and medicine, since many highly effective drugs have been developed on their basis. Most biological molecules, such as DNA and RNA, chlorophyll, hemoglobin, vitamins, and many others, contain a heterocycle as a key fragment. There are many heterocyclic compounds that are used in the treatment of common diseases (for example, derivatives of pyridine, oxazine, triazine, or benzimidazole, which have a wide variety of biological activities: antibacterial, antifungal, antiviral, anthelmintic, and other properties) [25,26,27,28,29,30,31,32,33,34]. Therefore, the study of the influence of a heterocycle structure on biological properties of compounds, including antiviral activity, is very promising and can solve a lot of medical problems, for example, the problem of drug resistance [35].

In this work, the antiviral activity of compounds **13** and **15** was investigated. We determined the inhibitory activity towards influenza virus strains with different antigenic formulae: A/Almaty/8/98 (H3N2); A/Vladivostok/2/09 (H1N1). The chemical therapeutic index (CTI) was determined at concentrations of compounds **13** and **15** from 0.0016% to 0.2%, which corresponded to doses of 0.003–0.4 mg per chicken embryo (0.06–8 mg/kg) (Table 2).

It was found that compound **15** did not show pronounced virus-inhibitory properties. However, compound **13** had a high CTI value, which is comparable to commercial drugs Tamiflu and Remantadine. Hence, the Schiff base 13 can be considered as a perspective anti-influenza drug candidate.

**Figure 6:**
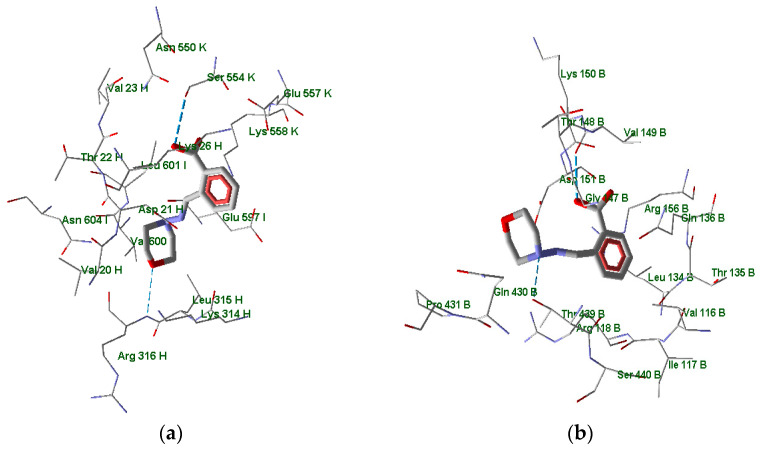
The best docking poses of compound **13** in the binding sites of 1930 Swine H1 hemagglutinin (PDB: 1RUY) (**a**) and Neuraminidase of 1918 H1N1 strain (PDB: 3BEQ) (**b**). The residues within 3 Å from each pose are visible. Hydrogen bonds are shown in blue dashed lines. The binding sites were preliminary located using AutoLigand methodology.

In order to evaluate affinities of active compound **13** to putative biotargets, we performed a docking study using the protein models of 1930 Swine H1 Hemagglutinin and Neuraminidase of 1918 H1N1 strain (PDB structures 1RUY [36] and 3BEQ [37], respectively). For these structures, the information on binding site location is not present in the Protein Data Bank. Hence, we undertook the search for binding sites with the use of AutoLigand methodology [38] implemented in AMDock 1.5.2 software. AutoLigand explores the space surrounding the protein and finds pockets with high probabilities of binding the investigated ligands. Within the found locations, the Autodock Vina program built in AMDock 1.5.2 was applied to calculate the docking poses of compound **13** and estimate the binding energies E_b_. The sites with the more negative E_b_ values and the corresponding docking poses are shown in Figure 6.

**Figure 7:**
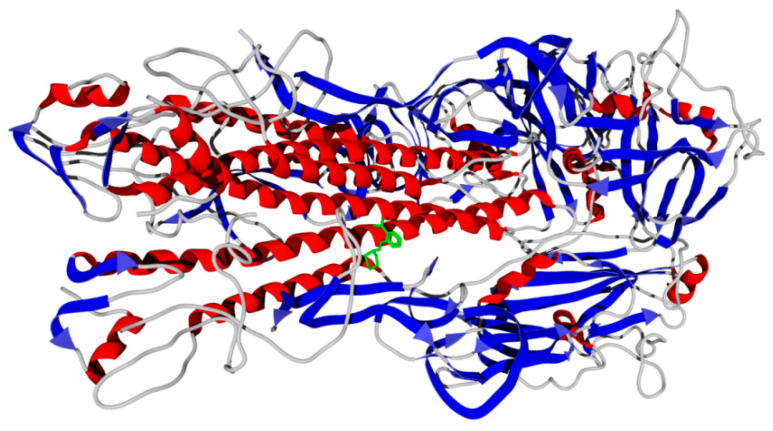
The secondary structure view of Swine H1 hemagglutinin (1RUY structure from Protein Data Bank) with the docking pose of ligand **13** (green). α-Helices and β-sheets are highlighted in red and blue, respectively.

According to the docking results, the molecule of compound **13** in the binding site is surrounded by the residues of three protein chains (H, I, and K) of a total of six chains contained in the 1RUY structure. The compound forms hydrogen bonds with the Arg316 residue of chain H with the participation of the morpholine oxygen atom. Also, a hydrogen bond is formed between the carboxyl group of ligand **13** and the Ser554 residue of chain K (Figure 6a). The nitrogen atom of the terminal ammonium group in Lys558K residue is positioned approximately 4.4 Å from the center of compound **13**’s benzene ring. Hence, there is a π-cation interaction between these molecular fragments. The location of the binding site in the entire 1RUY structure is shown in Figure 7.

**Figure 8:**
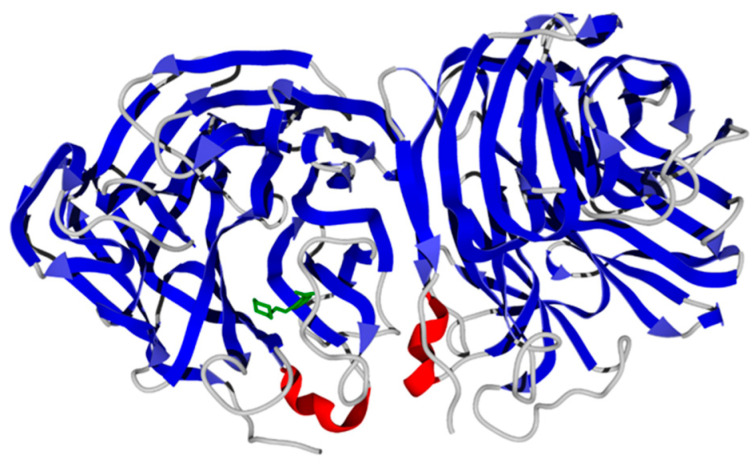
The secondary structure view of H1N1 neuraminidase (3BEQ structure from Protein Data Bank) with the docking pose of ligand **13** (green).

The docking of molecule **13** to H1N1 neuraminidase gave the best pose within the site located in Chain B of the 3BEQ structure (Figure 8). The morpholine and carboxyl moieties of the ligand are anchored by hydrogen bonds to residues Thr439 and Val149, respectively (Figure 6b). Again, the center of the benzene ring of compound **13** is in proximity (~4.5 Å) to the positively charged guanidine group of Arg156 residue, indicating the π-cation interaction between the ligand and the neuraminidase macromolecule.

The binding energies calculated with AutoDock Vina for compound **13** interaction with 1930 Swine H1 Hemagglutinin and Neuraminidase of 1918 H1N1 strain equal −6.6 and −6.7 kcal/mol, respectively, which corresponds to a micromolar range of affinity. Thus, the interaction of compound **13** with the investigated putative biotargets can be a rationale for its antiviral activity.

We have also studied the cytotoxic activity of 2-((morpholinoimino)methyl)benzoic acid **13** with respect to larvae of Artemia salina (Leach) crustaceans under in vitro cultivation conditions. The cytotoxicity of the sample was evaluated in the survival test of larvae of Artemia salina (Leach) crustaceans. The experiments were carried out on two-day-old larvae. The tests were performed using a ready-made sample, as well as positive and negative controls with dactinomycin (actinomycin D, LD_50_ = 57.5 mcg/mL), which has antitumor (cytotoxic) activity, and dimethyl sulfoxide used for the dilution of the test sample. A statistical analysis of the results was carried out using the FNI computer program.

It was found that compound **13** exhibits moderate cytotoxic activity (LD_50_ = 56.0 mcg/mL) against the larvae of Artemia salina (Leach) crustaceans.

The antimicrobial activity was studied on a sample of 2-((morpholinoimino)methyl)benzoic acid **13** towards strains of Gram-positive bacteria *Staphylococcus aureus*, *Bacillus subtilis*, Gram-negative bacteria *E. coli*, *Pseudomonas aeruginosa*, and the yeast fungus *Candida albicans* by means of diffusion into agar (wells) and serial dilution. The obtained results are shown in Table 3. The results of determining the minimum suppressive concentration (MSC) in relation to the reference microorganisms are presented in Table 4.

The results of the study showed that compound **13** had pronounced antibacterial activity against the Gram-negative strain *Escherichia coli* ATCC 25922; the minimum suppressive concentration (MSC) was 6.3 mcg/mL. Hydrazone **13** also exhibited moderate antimicrobial activity against the Gram-positive test strain *Staphylococcus aureus* ATCC 6538.

## 3. Materials and Methods

The parameters used for High-Performance Liquid Chromatography–Mass Spectrometry (HPLC-MS) analysis. Here is a breakdown of some of the key parameters mentioned: an Agilent 1260 Infinity II chromatograph («Agilent Technologies», Santa Clara, CA, USA, 2015) was coupled with an Agilent 6545 LC/Q-TOF high-resolution mass spectrometer («Agilent Technologies», USA, 2015). Ionization Source: Dual AJS ESI ionization source operating in positive ion mode.

Operating Parameters: Capillary voltage: 4000 V; Spray pressure: 20 psi inch; Drying gas flow rate: 10 L/min; Gas temperature: 325 °C; Gas flow in the shell: 12 L/min; Protective gas temperature: 400 °C; Nozzle voltage: 0 V; Fragmenter voltage: 180 V; Skimmer voltage: 45 V; RF octopole voltage: 750 V. Mass spectra with LC accuracy/MS were recorded in the range of 100–1000 *m*/*z*, and the scanning speed was 1.5 m/s. Chromatographic separation was carried out on ZORBAX RRHD Eclipse Plus C18 columns (2.1 × 50 mm, particle size 1.8 µm). The column temperature was maintained at 35 °C during the analysis. The mobile phase was formed by eluents A and B. In the positive ionization mode, 0.1% formic acid solution in deionized water was used as eluent A, and 0.1% formic acid solution in acetonitrile was used as eluent B. Chromatographic separation was performed during elution according to the following scheme: 0–10 min 95% A, 10–13 min 100% B, and 13–15 min 95% A. The flow of the mobile phase was maintained at 400 µL/min during the analysis. In all experiments, the sample input volume was 1 µL. The sample was prepared by dissolving the entire sample (in 1000 µL) in methanol (for HPLC). The dilution of the sample was carried out immediately before the analysis. The registered data were processed in the Agilent MassHunter 10.0 software.

The ^1^H and ^13^C NMR spectra were recorded on a JNM-ECA Jeol 400 (JEOL Ltd., Tokyo, Japan, 2008; frequencies of 399.78 and 100.53 MHz, respectively) spectrometer (frequency 399.78 and 100.53 MHz, respectively) using DMSO-d_6_ and CDCl_3_ solvents. Chemical shifts were measured relative to the signals of residual protons or carbon atoms of a deuterated solvent. IR spectra were recorded on an IR Fourier spectrometer FSM 1201 («Infraspec» LLC, St. Petersburg, Russia) in KBr tablets in the region of 400–4000 cm^−1^. The elemental analysis (C, H, and N) was performed on the EuroVector PE2400 SERIES II Elemental Analyser device («Eurovector», S.p.A., Rome, Italy, 2019). Melting temperatures were determined using the SMP10 device («Barloworld Scientific», Staffordshire, UK, 2017). TLC analysis was performed on Silufol UV-254 plates (Serva Feinbiochemica GmbH@Co, Prague, Czech Republic, 2022) with iodine vapors visualization.

The X-ray diffraction study of compound **15** was performed on a Bruker Optik GmbH (Bruker corp., Ettlingen, Germany), (MoK_α_, graphite monochromator, ω-scanning, 2.27° ≤ θ ≤ 27.51°) at a temperature of 296 K. In total, 21,510 reflections were captured, including 3147 independent ones (R_int_ = 0.0322). Monoclinic crystals, a = 9.9203(3), b = 8.7798(3), c = 15.9274(5) Å, β = 98.757(1)°, V = 1371.08(8) Å3, Z = 4 (C_14_H_16_N_2_O_4_), spatial group P_21/n_, d = 1.338 g/cm^3^, µ = 0.099 mm^−1^. The initial array of measured intensities was processed and absorption was taken into account using the SAINT [39] and SADABS [40] programs (multi-scan, T_min_ = 0.931, T_max_ = 0.983) [41].

The structure was deciphered using the direct method. The positions of nonhydrogen atoms were refined in an anisotropic approximation by the full-matrix least squares method. Hydrogen atoms were placed in geometrically calculated positions, and their coordinates were refined in an isotropic approximation with fixed positional and thermal parameters (the “rider” model). The calculations used 2562 reflections of independent reflections with I ≥ 2σ(I), and the number of specified parameters was 182. The final divergence factors were as follows: R_1_ 0.0413; _W_R_2_ 0.1137 (for reflections with I ≥ 2σ(I)); R_1_ 0.0525; _W_R_2_ 0.1247 (for all reflections); and GooF = 1.059. Residual density peaks: Δρ = 0.227 and −0.161 e/Å^3^. The structure was deciphered and refined according to the programs “SHELXT 2014/5” [41] and “SHELXL-2018/3” [42]. The X-ray data in the form of a CIF file were deposited at the Cambridge Crystal Structure Data Center (CCDC 2358257).

### 3.1. Synthesis of Hydrazones 8–13

N-aminomorpholine (0.01 mol) was dissolved in 5 mL of 2-propanol, and the corresponding aldehyde (0.011 mol) was added in 15 mL of 2-propanol. The mixture was refluxed for 3 h and cooled to room temperature. The resulting precipitate was filtered and recrystallized from 2-propanol.

**N-**(**2-bromo-3-phenylallylidene**)**morpholine-4-amine** (**8**)**.** Colorless powder, yield 42.6%, melting point 118–120 °C. IR spectrum (KBr), ν, cm^−1^: 1566 (C=N), 1450, 1492 (arom.). NMR ^1^H (CDCl_3_), δ, ppm., (J, Hz): 3.13–3.35 m (4H, H^3ax,5ax,3eq,5eq^), 3.82–4.03 m (4H, H^2ax,6ax^,^2eq,6eq^), 7.09–7.13 m (1H, H^10^), 7.27–7.39 m (4H, H^8,13,15,14^), 7.70–7.76 m (2H, H^12,16^). NMR ^13^C (CDCl_3_), δ_C_, ppm.: 51.95 (C^3,5^), 66.45 (C^2,6^), 121.54 (C^9^), 128.30 (C^12,16^), 128.49 (C^14^), 129.67 (C^13,15^), 132.51 (C^10^), 135.23 (C^8^), 135.50 (C^11^). COSY NMR: H^3,5^→H^2,6^, H^14^→H^13,15^, H^13,15^→H^12,16^. HMQC NMR: H^3,5^→C^3,5^, H^2,6^→C^2,6^, H^12,16^→C^12,16^, H^13,15^→C^13,15^, H^14^→C^14^, H^10^→C^10^, H^8^→C^8^. HMBC NMR: H^3,5^→C^2,6^; H^10^→C^9^, C^13,15^, C^11^; H^14^→C^12,16^, C^11^; H^12,16^.

**N-**(**4-**(**styryl**)**benzylidene**)**morpholine-4-amine** (**9**)**.** Light-yellow powder, yield 91.9%, melting point 219–222 °C. IR spectrum (KBr), ν, cm^−1^: 1697 (C=N), 1601, 1562 (arom.). NMR ^1^H (CDCl_3_), δ, ppm., (J, Hz): 3.12–3.19 m (4H, H^3ax,5ax,3eq,5eq^), 3.82–3.89 m (4H, H^2ax,6ax,2eq,6eq^), 7.02–7.64 m (11H, H^11,13,18,22,10,14,15,16,19,20,21^).NMR ^13^C (CDCl_3_), δC, ppm.: 51.79 (C^3,5^), 66.52 (C^2,6^), 126.56 (C^11,13^), 126.77 (C^18,22^), 135.25 (C^17^), 135.88 (C^12^), 137.33 (C^8^), 27.76, 128.27, 128.74 (C^10,14,19,20,21,6^). COSY NMR: H^3,5^→H^2,6^. HMQC NMR: H^3,5^→C^3,5^, H^2,6^→C^2,6^, H^18,22^→C^18,22^, H^11,13^→C^11,16^. Found, %: C 78.05; H 6.62; N 5.73. C_19_H_20_N_2_O. Calculated, %: C 78.05; H 6.90; N 9.58.

**4-bromo-2-**((**morpholinoimino**)**methyl**)**phenol** (**10**)**.** Colorless powder, yield 34.1%, melting point 143–146 °C. IR spectrum (KBr), ν, cm^−1^: 1674 (C=N), 1624, 1612 (arom.). NMR ^1^H (CDCl_3_), δ, ppm., (J, Hz): 3.10–3.15 m (4H, H^3ah,5ax,3eq,5eq^), 3.82–3.89 m (4H, H^2ax,6ax,2eq,6eq^), 6.75–6.84 m (1H, H^8^), 7.19–7.28 m (2H, H^8,12^), 7.53–7.60 m (1H, H^14^). 11.40–11.46 m (1H, H^15^). NMR ^13^C (CDCl_3_), δ_C_, ppm.: C^3,5^), 66.17 (C^2,6^), 131.81 (C^8^), 110.76 (C^13^), 118.53 (C^11^), 120.64 (C^9^), 132.29 (C^12^), 138.80 (C^14^), 156.67 (C^10^). COSY NMR: H^3,5^→H^2,6^, H^11^→H^12^. HMQC NMR: H^3,5^→C^3,5^, H^2,6^→C^2,6^, H^11^→C^11^, H^12^→C^12^, H^14^→C^14^. HMBC NMR: H^3,5^→C^2,6^; H^12^→C^14^, C^10^; H^14^→C^9^, C^12^, C^10^; H^15^→C^11^, C^12^, C^10^. Found, %: C 45.05; H 4.41; N 10.32. C_7_H_5_BrN_2_O_2_. Calculated, %: C 46.34; H 6.60; N 9.82.

**N-**(**pyridine-4-ilmethyl**)**morpholine-4-amine** (**11**)**.** Colorless powder, yield 36%, melting point 85 °C. IR spectrum (KBr), ν, cm^−1^: 1601 (C=N), 1539, 1581 (arom.). NMR ^1^H (CDCl_3_), δ, ppm (J, Hz): 3.15–3.18 m (4H, H^3ax,5ax,3eq,5eq^), 3.80–3.82 m (4H, H^2ax,6ax,2eq,6eq^), 7.30–7.38 m (3H, H^10,14,8^), 8.47–8.49 m (2H, H^11,14^). NMR ^13^C (CDCl_3_), δ_C_, m.d.: 51.25 (C^3.5^), 66.30 66.30 (C^2,6^), 131.66 (C^8^), 120.21 (C^10,14^), 131.66 (C^8^), 143.38 (C^9^), 150.10 (C^11,13^). COSY NMR: H^3,5^→H^2,6^, H^10,14^→H^11,13^. HMQC NMR: H^3,5^→C^3,5^, H^2,6^→C^2,6^, H^10,14^→C^10,14^, H^8^→C^8^, H^11,13^→C^11,13^. HMBC NMR: H^3,5^→C^2,6^; H^10,14^→C^9^; H^8^→C^10,14^, C^11,13^; H^11,15^→C^10,14^, C^9^. Found, %: C 61.31; H 6.60; N 21.12. C_10_H_13_N_3_O. Calculated, %: C 62.81; H 6.85; N 21.97.

**2-ethoxy-4-**((**morpholinoimino**)**methyl**)**phenol** (**12**)**.** Light-brown powder, yield 67.1%, melting point 142–145 °C. IR spectrum (KBr), ν, cm^−1^: 1581 (C=N), 1539, 1550 (arom.). NMR ^1^H (CDCl_3_), δ, ppm, (J, Hz): 1.39–1.43 m (3H, H-17,17,17), 3.10–3.18 m (4H, H^3ax,5ax,3eq,5eq^), 3.65–3.84 m (4H, H^2ax,6ax,2eq,6eq^), 4.09–4.14 m (2H, H-16,16), 6.11 br. s ((1H, H^18^), 6.85–6.94 m (2H, H^13,14^), 7.25 c (1H, H^10^), 7.44–7.53 m (1H, H^8^). NMR ^13^C (CDCl3), δ_C_, ppm: 14.93 (C^17^), 52.39 (C^3,5^), 66.60 (C^2,6^), 64.47 (C^16^), 107.93 (C^10^), 114.26 (C^13^), 121.25 (C^14^), 128.49 (C^9^), 146.40 (C^12^), 146.69 (C^11^), 137.47 (C^8^). COSY NMR: H^3,5^→H^2,6^, H^17^→H^16^. HMQC NMR: H^3,5^→C^3,5^, H^2,6^→C^2,6^, H^16^→C^16^, H^10^→C^10^, H^13^→C^13^, H^14^→C^14^, H^8^→C^8^. HMBC NMR: H^17^→C^16^; H^3,5^→C^2,6^; H^16^→C^17^, C^11^; H^8^→C^10^, C^14^, C^9^. Found, %: C 61.26; H 7.09; N 10.92. C_13_H_18_N_2_O_3_. Calculated, %: C 62.38; H 7.25; N 11.19.

**2-**((**Morpholinoimino**)**methyl**)**benzoic acid** (**13**)**.** Colorless powder, yield 91.4%, melting point 123–124 °C. IR spectrum (KBr), v, cm^−1^: 1698 (C=N), 1470, 1550 (arom.). ^1^H NMR spectrum (DMSO-d6), δ, ppm, (J, Hz): 3.05 d (4H, H3,3,5,5, 3J 3.4), 3.72 d (4H, H2,2,6, 6, 3J 3.4), 7.29–7.34 m (1H, H^11^), 7.44–7.94 m (1H, H^12^), 7.77–7.80 m (1H, H^10^), 7.85–7.88 (1H, H^13^), 8.31–8.32 m (1H, H^8^). ^13^C NMR spectrum (DMSO-d6), δ_C_, ppm: 51.93 (C^3,5^), 66.16 (C^2,6^), 126.22 (C^13^), 128.10 (C^11^), 130.78 (C^10^), 132.13 (C^12,14^), 134.70 (C^9^), 136.72 (C^8^), 168.99 (C^15^). COSY NMR spectrum: H^3.5^→H^2.6^, H^11^→H^12^, H^11^→H^10^, H^12^→H^13^. HMQC NMR spectrum: H^3.5^→C^3.5^, H^2.6^→C^2.6^, H^11^→C^11^, H^12^→C^12^, H^10^→C^10^, H^13^→C^13^, H^8^→C^8^. Found, %: C 61.96; H 6.12; N 11.71. C_12_H_14_N_2_O_3_. Calculated, %: C 61.53; H 6.02; N 11.96. Mass spectrum, *m*/*z*, (I,%): 235.116 (100) [M+H]+.

**2-Morpholino-3-oxoisoindolin-1-yl acetate** (**15**)**.** A mixture of 0.95 g of hydrazone **13** and 2.5 mL Ac_2_O was heated to dissolution, boiled for 3 min, and cooled. A mixture of 5 mL MeOH and 15 mL H_2_O was added. The obtained oil gradually crystallized when cooled with ice and rubbed with a stick. The precipitate was filtered, washed with petroleum ether, and dried. A colorless powder with a yellowish tinge was isolated. Yield 0.76 g (67.8%), melting point 168–170 °C. IR spectrum (KBr), ν, cm^−1^: 1745 (C=O cycle.), 1710 (C=O), 1395 (arom.). ^1^H NMR spectrum (DMSO-d6), δ, ppm, (J, Hz): 2.10 s (3H, H20,20,20), 3.18–3.23 m (2H, H^3ax,5ax^), 3.29–3.35 m (2H, H^3eq,5eq^), 3.58–3.62 m (4H, H^2ax,6xa,2eq,6eq^), 7.01 s (1H, H^8^), 7.50–7.51 m (1H, H^13^), 7.55–7.58 m (1H, H^11^), 7.62–7.64 m (1H, H^12^), 7.65–7.67 m (H^10^). ^13^C NMR spectrum (DMSO-d6), δ_C_, ppm: 21.34 (C^20^), 52.43 (C^3,5^), 67.13 (C^2,6^), 81.22 (C^8^), 123.6 (C^12^), 124.49 (C^13^), 130.92 (C^11^), 131.31 (C^14^), 133.57 (C^10^), 140.26 (C^9^), 166.18 (C^15^), 170.95 (C^17^). COSY NMR spectrum: H^3ax,5ax^→H^3eq,5eq^, H^3ax,5ax^→H^2ax,6ax^, H^3eq,5eq^→H^2eq,6eq^, H^13^→H^12^, H^11^→H^10^. HMQC NMR spectrum: H^20^→C^20^, H^3ax,5ax^→C^3,5^, H^3eq,5eq^→C^3,5^, H^2ax,2eq,6ax,6eq^→C^2,6^, H^11^→C^11^, H^12^→C^12^, H^10^→C^10^, H^13^→ C^13^, H^8^→C^8^. HMBC NMR spectrum: H^20^→C^17^; H^8^→C^14^, C^9^, C^15^, C^17^. Found, %: C 60.87; H 5.89; N 9.80. C_14_H_16_N_2_O_4_. Calculated, %: C 60.86; H 5.84; N 10.14. Mass spectrum, *m*/*z*, (Iotn, %): 276.07 (100) [M+H]+.

### 3.2. Molecular Docking

The 3D molecular model of compound **13** (ligand) was prepared using ChemOffice 16 software. The protein structures of 1930 Swine H1 Hemagglutinin and Neuraminidase of 1918 H1N1 strain were downloaded from the Protein Data Bank (PDB). The PDB codes of the structures were 1RUY and 3BEQ, respectively. The ligand and protein structures were imported into AMDock suite of programs (Version 1.5.2) [43] and converted to PDBQT format. The protonation states were adjusted to pH 7.4. The locations of the potential binding sites in the protein structures were found with the use of the AutoLigand method. The docking of compound **13** into the binding sites was performed with the default options of the AutoDock Vina module embedded in AMDock. The obtained docking poses with the most negative binding energy for each biotarget (1RUY and 3BEQ) were analyzed and visualized using Molegro 6.0 software.

### 3.3. Research of Biological Activity

The MTT test was used to evaluate the cytotoxicity of the compounds. The MTT test is based on the ability of living cell dehydrogenases to reduce the uncolored forms of 3-4,5-dimethylthiazol-2-yl-2,5-diphenylterarazole (MTT reagent) to blue crystalline pharmazan, soluble in dimethyl sulfoxide. In our experiments, both positive and negative controls were used:

Known cytotoxic agents were used as positive controls.

Untreated cells and cells treated with solvent (e.g., DMSO) at appropriate concentrations were used as negative controls to eliminate possible solvent influences on the results.

Hydrazone concentrations varied depending on the type of test performed:

To test cytotoxic activity, hydrazone concentrations were in the range of 0.03–1%.

In studies of antiviral activity, the maximum concentration of hydrazones was 1 mg/100 μL.

Concentrations ranging from 0.003% to 0.4% were used to evaluate virus inhibitory activity.

## 4. Conclusions

This research describes a chemical synthesis study involving the reaction of N-aminomorpholine with functionally substituted benzaldehydes and 4-pyridinaldehyde in isopropyl alcohol to form hydrazones. The synthesized compounds were then characterized using various spectroscopic techniques like ^1^H and ^13^C NMR, as well as advanced techniques like COSY, HMQC, and HMBC. This study highlights the importance of structural characterization in identifying novel compounds with valuable biological activities. The compound 2-((morpholinoimino)methyl)benzoic acid was identified to have significant antiviral properties, comparable to the well-known antiviral drugs Tamiflu and Remantadine. This finding suggests potential applications of this compound as an antiviral agent.

## Figures and Tables

**Table 1:** The synthesis of new N-aminomorpholine hydrazones.

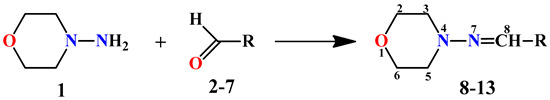	
**Compound**	**R**
**2**, **8**	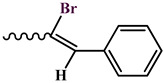
**3**, **9**	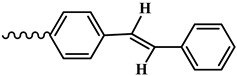
**4**, **10**	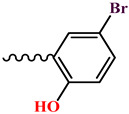
**5**, **11**	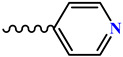
**6**, **12**	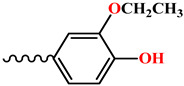
**7**, **13**	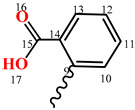

**Table 2:** The viral inhibitory activity of compounds **13** and **15** with respect to influenza viruses.

Compound	Chemical Therapeutic Index	
	A/Almaty/8/98 (H3N2)	A/Vladivostok/2/09 (H1N1)
**13**	25.0	20.0
**15**	<1.0	<1.0
**Tamiflu**	29.9	30
**Remantadine**	10.3	11

**Table 3:** The antimicrobial activity of compound **13** compared to some other antimicrobial agents.

Compound	*Staphylococcus* *aureus* *ATCC 6538*	*Bacillus* *subtilis* *ATCC 6633*	*Escherichia* *coli* *ATCC 25922*	*Pseudomonas* *aeruginosa* *ATCC 27853*	*Candida* *albicans ATCC 10231*
2-((morpholino-amino) methyl)-benzoic acid (**13**)	18 ± 0.1	15 ± 0.1 *	21 ± 0.1 *	-	14 ± 0.1
Benzylpenicillin sodium salt	16 ± 0.1	14 ± 0.1	15 ± 0.1	-	-
Gentamicin	24 ± 0.1	21 ± 0.2	26 ± 0.1	27 ± 0.1	-
Nystatin	-	-	-		21 ± 0.2

Note: *—the significance of the differences is *p* < 0.05 compared to the control group.

**Table 4:** Minimum suppressive concentration (MSC) of compound **13** in relation to reference test strains.

Compound	MSC (mcg/mL)				
	*Staphylococcus* *aureus* *ATCC 6538*	*Bacillus* *subtilis* *ATCC 6633*	*Escherichia* *coli* *ATCC 25922*	*Pseudomonas* *aeruginosa* *ATCC 27853*	*Candida* *albicans ATCC 10231*
2-((morpholino-amino) methyl)-benzoic acid (**13**)	12.5	25	6.3	-	50

## Data Availability

The data that support the findings of this study are available within the article and the Supplementary Materials. Further data are available from the corresponding author upon reasonable request.

## Supplementary Materials

The following supporting information can be downloaded at: https://www.mdpi.com/article/10.3390/molecules29153606/s1.
