# Supplementary material for: Synthesis and Biological Activity of New Hydrazones Based on N-Aminomorpholine
Source: Molecules. 2024 Jul 30;29(15):3606. doi: 10.3390/molecules29153606 (PMC11314140; doi:10.3390/molecules29153606)
Supplement: Supplementary file 1 [file molecules-29-03606-s001.zip › molecules-3112167-supplementary.pdf]

Supporting information

Synthesis and biological activity of new hydrazones based on N-aminomorpholine

Table of contents

Experimental 1

General Information..1

Experimental Procedures..2

Spectroscopic and physical data 3

Copies of NMR Spectra of Products 4

Copies of MS Spectra of Products..12

X-Ray Structural Study of Product..13

EuroVector Elemental Analyser 14

Experimental

General Information

Samples were analyzed by HPLC-MS on an Agilent 1260 Infinity II chromatograph coupled to an Agilent 6545 LC/Q-TOF high-resolution mass spectrometer with a Dual AJS ESI ionization source operating in positive ion mode, using the following parameters: capillary voltage: 4000 V; Spray Pressure: 20 (psi) psi; drying gas: 10 l/min; gas temperature: 325 ºC; gas flow in the shell: 12 l/min; shielding gas temperature: 400 C; nozzle voltage: 0 V, fragmenter voltage: 180 V; skimmer voltage: 45 V; Octopole RF: 750 V. Mass spectra with LC/MS accuracy were recorded in the range of 100-1000 m/z scanning speed 1.5 spectra/s.

Chromatographic separation was carried out on columns: ZORBAX RRHD Eclipse Plus C18 (2.1 x 50 mm, particle size 1.8 μm). The column temperature was maintained at 35°C during the analysis. The mobile phase was formed by eluents A and B. In the positive ionization mode, a 0.1% solution of formic acid in deionized water was used as eluent A, and a 0.1% solution of formic acid in acetonitrile was used as eluent B. Chromatographic separation was performed with elution according to the following scheme: 0-10 min 95% A, 10-13 min 100% B, 13-15 min 95% A. The mobile phase flow was maintained at 400 μl/min throughout the analysis. In all experiments, the sample injection volume was 1 μL. The sample was prepared by dissolving the entire sample (in 1000 μl) in methanol (for HPLC). Sample dilution was carried out immediately before analysis.The recorded data was processed in Agilent MassHunter 10.0 software.

Experimental Procedures

N-(2-bromo-3-phenylallidene)morpholine-4-amine (8), (fig. S1- S5). N-aminomorpholine (0.01 mol) was dissolved in 5 ml of 2-propanol and the α-bromocinnamaldehyde (0.011 mol) was added in 15 ml of 2-propanol. The mixture was boiled with a return refrigerator for 3 hours, and then cooled to room temperature. The resulting precipitate was filtered and recrystallized from 2-propanol. White powder, yield 42.6%, melting point 118-120°C.

N-(4-(styryl)benzylidene)morpholine-4-amine (9), (fig. S6- S10). N-aminomorpholine (0.01 mol) was dissolved in 5 ml of 2-propanol and the 4-styrylbenzaldehyde (0.011 mol) was added in 15 ml of 2-propanol. The mixture was boiled with a return refrigerator for 3 hours, and then cooled to room temperature. The resulting precipitate was filtered and recrystallized from 2-propanol. Light yellow powder, yield 91.9%, melting point 219-222°C.

4-bromo-2-((morpholinoamino)methyl)phenol (10), (fig. S11- S15). N-aminomorpholine (0.01 mol) was dissolved in 5 ml of 2-propanol and the 5-bromo-2-hydroxybenzaldehyde (0.011 mol) was added in 15 ml of 2-propanol. The mixture was boiled with a return refrigerator for 3 hours, and then cooled to room temperature. The resulting precipitate was filtered and recrystallized from 2-propanol. White powder, yield 34.1%, melting point 143-146°C.

N-(pyridine-4-ilmethyl)morpholine-4-amine (11), (fig. S16- S20). N-aminomorpholine (0.01 mol) was dissolved in 5 ml of 2-propanol and the 4-pyridinecarboxaldehyde (0.011 mol) was added in 15 ml of 2-propanol. The mixture was boiled with a return refrigerator for 3 hours, and then cooled to room temperature. The resulting precipitate was filtered and recrystallized from 2-propanol. White powder, yield 36%, melting point 85°C.

2-ethoxy-4-((morpholinoamino)methyl)phenol (12), (fig. S21- S25). N-aminomorpholine (0.01 mol) was dissolved in 5 ml of 2-propanol and the 3-ethoxy-4-hydroxbenzaldehyde (0.011 mol) was added in 15 ml of 2-propanol. The mixture was boiled with a return refrigerator for 3 hours, and then cooled to room temperature. The resulting precipitate was filtered and recrystallized from 2-propanol. Light brown powder, yield 67.2%, melting point 142-145°C.

2-((Morpholinoamino)methyl)benzoic acid (13), (fig. S26- S29). N-aminomorpholine (0.01 mol) was dissolved in 5 ml of 2-propanol and the 2-carboxybenzaldehyde (0.011 mol) was added in 15 ml of 2-propanol. The mixture was boiled with a return refrigerator for 3 hours, and then cooled to room temperature. The resulting precipitate was filtered and recrystallized from 2-propanol. White powder, yield 91.4%, melting point 123-124°C.

2-Morpholino-3-oxoisoindoline-1-yl acetate (15), (fig. S30- S34). A mixture of 0.95 g of hydrazone **13** and 2.5 ml Ac_2O was heated to dissolution and boiled for 3 minutes, cooled and a mixture of 5 ml MeOH and 15 ml H_2O was added. The released oil gradually crystallized when cooled with ice and rubbed with a stick. The sediment was filtered, washed with petroleum ether and dried. A white powder with a yellowish tinge was obtained with a temperature of 168-170°C. The yield is 0.76 g (67.8%).

Spectroscopic and physical data

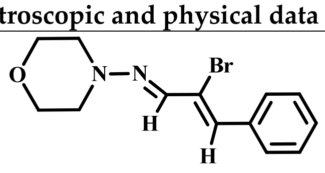 (*E*)-*N*-((*Z*)-2-bromo-3-phenylallylidene)morpholin-4-amine<br>Molecular Weight: 295,17	**N-(2-bromo-3-phenylallidene)morpholine-4-amine (8).** White powder, yield 42.6%, melting point 118-120°C.<br>IR spectrum (KBr), ν, cм$^{-1}$: 1566 (C=N), 1450, 1492 (arom.).NMR spectrum ^{1}H ($CDCl_3$), δ, ppm., (J, Hz): 3.13-3.35 m (4H, $H^{3ax,5ax,3eq,5eq}$), 3.82-4.03 m (4H, $H^{2ax,6ax,2eq,6eq}$), 7.09-7.13 m (1H, H^{10}), 7.27-7.39 m (4H, $H^{8,13,15,14}$), 7.70-7.76 m (2H, $H^{12,16}$). NMR spectrum ^{13}C ($CDCl_3$), δ_C, ppm.: 51.95 ($C^{3,5}$), 66.45 ($C^{2,6}$), 121.54 (C^9), 128.30 ($C^{12,16}$), 128.49 (C^{14}), 129.67 ($C^{13,15}$), 132.51 (C^{10}), 135.23 (C^8), 135.50 (C^{11}). COSY NMR spectrum: $H^{3,5}\rightarrow H^{2,6}$, $H^{14}\rightarrow H^{13,15}$, $H^{13,15}\rightarrow H^{12,16}$. HMQC NMR spectrum: $H^{3,5}\rightarrow C^{3,5}$, $H^{2,6}\rightarrow C^{2,6}$, $H^{12,16}\rightarrow C^{12,16}$, $H^{13,15}\rightarrow C^{13,15}$, $H^{14}\rightarrow C^{14}$, $H^{10}\rightarrow C^{10}$, $H^8\rightarrow C^8$. HMBC NMR spectrum: $H^{3,5}\rightarrow C^{2,6}$; $H^{10}\rightarrow C^9$, $C^{13,15}$, C^{11}; $H^{14}\rightarrow C^{12,16}$, C^{11}; $H^{12,16}$

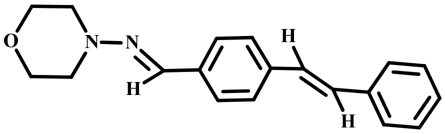<br>(*E*)-*N*-(4-((*E*)-styryl)benzylidene)morpholin-4-amine<br>Molecular Weight: 292,37	**N-(4-(styryl)benzylidene)morpholine-4-amine (9).** Light yellow powder, yield 91.9%, melting point 219-222°C.<br>IR spectrum (KBr), ν, cm^{-1}: 1697 (C=N), 1601, 1562 (arom.). NMR spectrum ^{1}H ($CDCl_3$), δ, ppm., (J, Hz): 3.12-3.19 m (4H, $H^{3ax,5ax,3eq,5eq}$), 3.82-3.89 m (4H, $H^{2ax,6ax,2eq,6eq}$), 7.02-7.64 m (11H, $H^{11,13,18,22,10,14,15,16,19,20,21}$).NMR spectrum ^{13}C ($CDCl_3$), δC, ppm.: 51.79 ($C^{3,5}$), 66.52 ($C^{2,6}$), 126.56 ($C^{11,13}$), 126.77 ($C^{18,22}$), 135.25 ($C^{17}$), 135.88 ($C^{12}$), 137.33 ($C^{8}$), 27.76, 128.27, 128.74 ($C^{10,14,19,20,21,6}$). COSY NMR spectrum: $H^{3,5}$→$H^{2,6}$. HMQC NMR spectrum: $H^{3,5}$→$C^{3,5}$, $H^{2,6}$→$C^{2,6}$, $H^{18,22}$→$C^{18,22}$, $H^{11,13}$→$C^{11,16}$. Found, %: C 78.05; H 6.62; N 5.73. $C_{19}H_{20}N_2O$. Calculated, %: C 78.05; H 6.90; N 9.58.
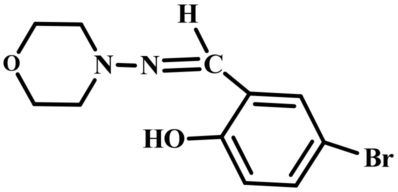<br>4-bromo-2-((morpholinoimino)methyl)phenol<br>Molecular Weight: 285,14	**4-bromo-2-((morpholinoamino)methyl)phenol (10).** White powder, yield 34.1%, melting point 143-146°C.<br>IR spectrum (KBr), ν, cm^{-1}: 1674 (C=N), 1624, 1612 (arom.). NMR spectrum ^{1}H ($CDCl_3$), δ, ppm., (J, Hz): 3.10-3.15 m (4H, $H^{3ah,5ax,3eq,5eq}$), 3.82-3.89 m (4H, $H^{2ax,6ax}$^{,2eq,6eq}), 6.75-6.84 m (1H, H^{8}), 7.19-7.28 m (2H, $H^{8,12}$), 7.53-7.60 m (1H, H^{14}).11.40-11.46 m (1H, H^{15}). NMR spectrum ^{13}C ($CDCl_3$), δ_C, ppm.: $C^{3,5}$), 66.17 ($C^{2,6}$), 131.81 (C^{8}), 110.76 (C^{13}), 118.53 (C^{11}), 120.64 (C^{9}), 132.29 (C^{12}), 138.80 (C^{14}), 156.67 (C^{10}). COSY NMR spectrum: $H^{3,5}$→$H^{2,6}$, H^{11}→H^{12}. HMQC NMR spectrum: $H^{3,5}$→$C^{3,5}$, $H^{2,6}$→$C^{2,6}$, H^{11}→C^{11}, H^{12}→C^{12}, H^{14}→C^{14}. HMBC NMR spectrum: $H^{3,5}$→$C^{2,6}$; H^{12}→C^{14}, C^{10}; H^{14}→C^{9}, C^{12}, C^{10}; H^{15}→C^{11}, C^{12}, C^{10}. Found, %: C 45.05; H 4.41; N 10.32. $C_7H_5BrN_2O_2$. Calculated, %: C 46.34; H 6.60; N 9.82.
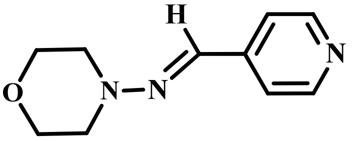<br>(*E*)-*N*-(pyridin-4-ylmethylene)morpholin-4-amine<br>Molecular Weight: 191,23	**N-(pyridine-4-ilmethyl)morpholine-4-amine (11).** White powder, yield 36%, melting point 85°C.<br>IR spectrum (KBr), ν, cm^{-1}: 1601 (C=N), 1539, 1581 (arom.). NMR spectrum ^{1}H ($CDCl_3$), δ, ppm (J, Hz): 3.15-3.18 m (4H, $H^{3ax,5ax,3eq,5eq}$), 3.80-3.82 m (4H, $H^{2ax,6ax}$^{,2eq,6eq}), 7.30-7.38 m (3H, $H^{10,14,8}$), 8.47-8.49 m (2H, $H^{11,14}$). NMR spectrum ^{13}C ($CDCl_3$), δ_C, m.d.: 51.25 ($C^{3.5}$), 66.30 66.30 ($C^{2,6}$), 131.66 (C^{8}), 120.21 ($C^{10,14}$), 131.66 (C^{8}), 143.38 (C^{9}), 150.10 ($C^{11,13}$). COSY NMR spectrum: $H^{3,5}$→$H^{2,6}$, $H^{10,14}$→$H^{11,13}$. HMQC NMR spectrum: $H^{3,5}$→$C^{3,5}$, $H^{2,6}$→$C^{2,6}$, $H^{10,14}$→$C^{10,14}$, H^{8}→C^{8}, $H^{11,13}$→$C^{11,13}$. HMBC NMR spectrum: $H^{3,5}$→$C^{2,6}$; $H^{10,14}$→C^{9}; H^{8}→$C^{10,14}$, $C^{11,13}$; $H^{11,15}$→$C^{10,14}$, C^{9}. Found, %: C 61.31; H 6.60; N 21.12. $C_{10}H_{13}N_3O$. Calculated, %: C 62.81; H 6.85; N 21.97.

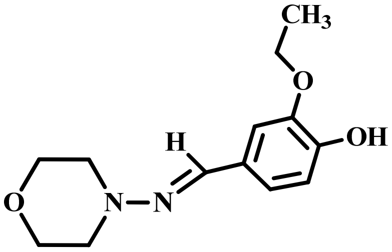 (*E*)-2-ethoxy-4-((morpholinoimino)methyl)phenol<br>Molecular Weight: 250,29	**2-ethoxy-4-((morpholinoamino)methyl)-phenol (12).** Light brown powder, yield 67.2%, melting point 142-145°C. IR spectrum (KBr), ν, cm^{-1}: 1581 (C=N), 1539, 1550 (arom.).NMR spectrum ^{1}H (CDCl$_3$), δ, ppm., (J, Hz): 1.39-1.43 m (3H, H-17,17,17), 3.10-3.18 м (4H, $H^{3ax,5ax,3eq,5eq}$), 3.65-3.84 м (4H, $H^{2ax,6ax,2eq,6eq}$), 4.09-4.14 м (2H, H-16,16), 6.11 br. s ((1H, H^{18}), 6.85-6.94 м (2H, $H^{13,14}$), 7.25 c (1H, H^{10}), 7.44-7.53 m (1H, H^{8}). NMR spectrum ^{13}C (CDCl3), δ_C, ppm.: 14.93 (C^{17}), 52.39 ($C^{3,5}$), 66.60 ($C^{2,6}$), 64.47 (C^{16}), 107.93 (C^{10}), 114.26 (C^{13}), 121.25 (C^{14}), 128.49 (C^{9}), 146.40 (C^{12}), 146.69 (C^{11}), 137.47 (C^{8}). COSY NMR spectrum: $H^{3,5}\rightarrow H^{2,6}$, $H^{17}\rightarrow H^{16}$. HMQC NMR spectrum: $H^{3,5}\rightarrow C^{3,5}$, $H^{2,6}\rightarrow C^{2,6}$, $H^{16}\rightarrow C^{16}$, $H^{10}\rightarrow C^{10}$, $H^{13}\rightarrow C^{13}$, $H^{14}\rightarrow C^{14}$, $H^{8}\rightarrow C^{8}$. HMBC NMR spectrum: $H^{17}\rightarrow C^{16}$; $H^{3,5}\rightarrow C^{2,6}$; $H^{16}\rightarrow C^{17}$, C^{11}; $H^{8}\rightarrow C^{10}$, C^{14}, C^{9}. Found, %: C 61.26; H 7.09; N 10.92. $C_{13}H_{18}N_2O_3$. Calculated, %: C 62.38; H 7.25; N 11.19.
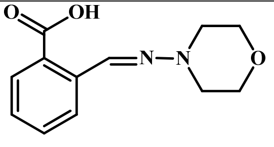 2-((morpholinoimino)methyl)benzoic acid<br>Molecular Weight: 234,25	**2-((Morpholinoamino)methyl)benzoic acid (13).** Спектр ЯМР ^{1}H (ДМСО-d$_6$), δ, м.д., (*J*, Гц): 3.05 д (4H, $H^{3,3,5,5}$, ^{3}J 3.4), 3.72 д (4H, $H^{2,2,6,6}$, ^{3}J 3.4), 7.29-7.34 м (1H, H^{11}), 7.44-7.94 м (1H, H^{12}), 7.77-7.80 м (1H, H^{10}), 7.85-7.88 (1H, H^{13}), 8.31-8.32 м (1H, H^{8}). Спектр ЯМР ^{13}C (ДМСО-d$_6$), δ_C, м.д.: 51.93 ($C^{3,5}$), 66.16 ($C^{2,6}$), 126.22 (C^{13}), 128.10 (C^{11}), 130.78 (C^{10}), 132.13 ($C^{12,14}$), 134.70 (C^{9}), 136.72 (C^{8}), 168.99 (C^{15}). Спектр ЯМР COSY: $H^{3,5}\rightarrow H^{2,6}$, $H^{11}\rightarrow H^{12}$, $H^{11}\rightarrow H^{10}$, $H^{12}\rightarrow H^{13}$. Спектр ЯМР HMQC: $H^{3,5}\rightarrow C^{3,5}$, $H^{2,6}\rightarrow C^{2,6}$, $H^{11}\rightarrow C^{11}$, $H^{12}\rightarrow C^{12}$, $H^{10}\rightarrow C^{10}$, $H^{13}\rightarrow C^{13}$, $H^{8}\rightarrow C^{8}$.
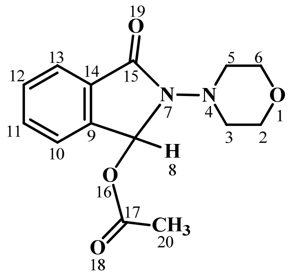 2-morpholino-3-oxoisoindolin-1-yl acetate<br>Molecular Weight: 276,29	**2-Morpholino-3-oxoisoindoline-1-yl acetate (15).** Спектр ЯМР ^{1}H (ДМСО-d$_6$), δ, м.д., (*J*, Гц): 2.10 c (3H, $H^{20,20,20}$), 3.18-3.23 м (2H, $H^{3ax,5ax}$), 3.29-3.35 м (2H, $H^{3eq,5eq}$), 3.58-3.62 м (4H, $H^{2ax,6xa,2eq,6eq}$), 7.01 c (1H, H^{8}), 7.50-7.51 м (1H, H^{13}), 7.55-7.58 м (1H, H^{11}), 7.62-7.64 м (1H, H^{12}), 7.65-7.67 м (H^{10}). Спектр ЯМР ^{13}C (ДМСО-d$_6$), δ_C, м.д.: 21.34 (C^{20}), 52.43 ($C^{3,5}$), 67.13 ($C^{2,6}$), 81.22 (C^{8}), 123.6 (C^{12}), 124.49 (C^{13}), 130.92 (C^{11}), 131.31 (C^{14}), 133.57 (C^{10}), 140.26 (C^{9}), 166.18 (C^{15}), 170.95 (C^{17}). Спектр ЯМР COSY: $H^{3ax,5ax}\rightarrow H^{3eq,5eq}$, $H^{3ax,5ax}\rightarrow H^{2ax,6ax}$, $H^{3eq,5eq}\rightarrow H^{2eq,6eq}$, $H^{13}\rightarrow H^{12}$, $H^{11}\rightarrow H^{10}$. Спектр ЯМР HMQC: $H^{20}\rightarrow C^{20}$, $H^{3ax,5ax}\rightarrow C^{3,5}$, $H^{3eq,5eq}\rightarrow C^{3,5}$, $H^{2ax,2eq,6ax,6eq}\rightarrow C^{2,6}$, $H^{11}\rightarrow C^{11}$, $H^{12}\rightarrow C^{12}$, $H^{10}\rightarrow C^{10}$, $H^{13}\rightarrow C^{13}$, $H^{8}\rightarrow C^{8}$. Спектр ЯМР HMBC: $H^{20}\rightarrow C^{17}$; $H^{8}\rightarrow C^{14}$, C^{9}, C^{15}, C^{17}.

Copies of NMR Spectra of Products

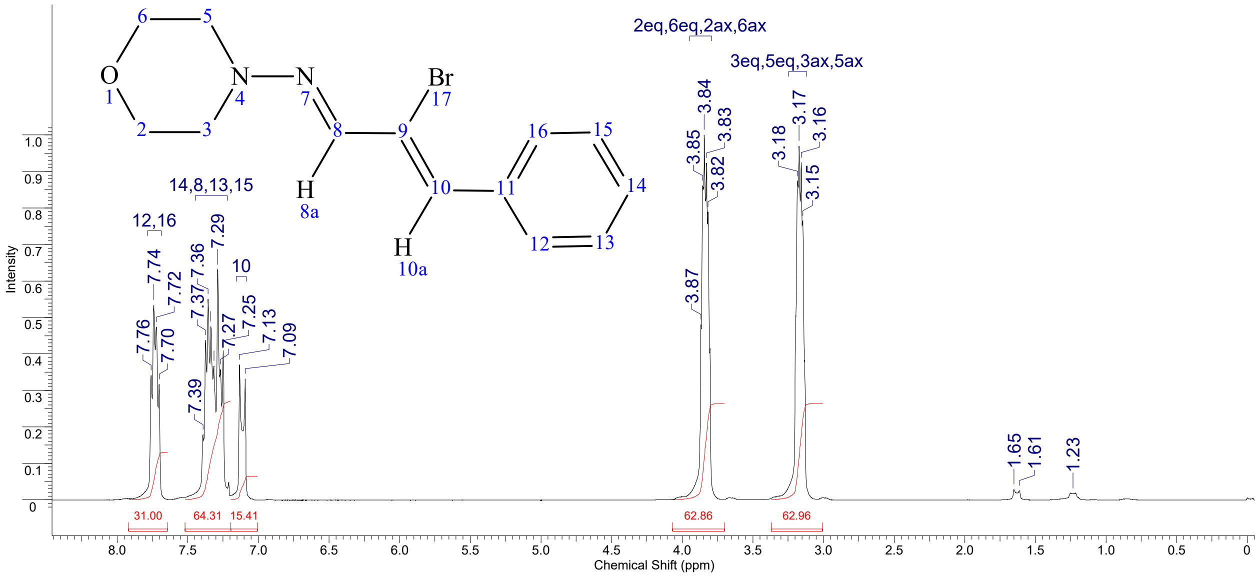

Fig. S1. ^{1}H (399.78 MHz, $CDCl_3$) NMR Spectra of **8**

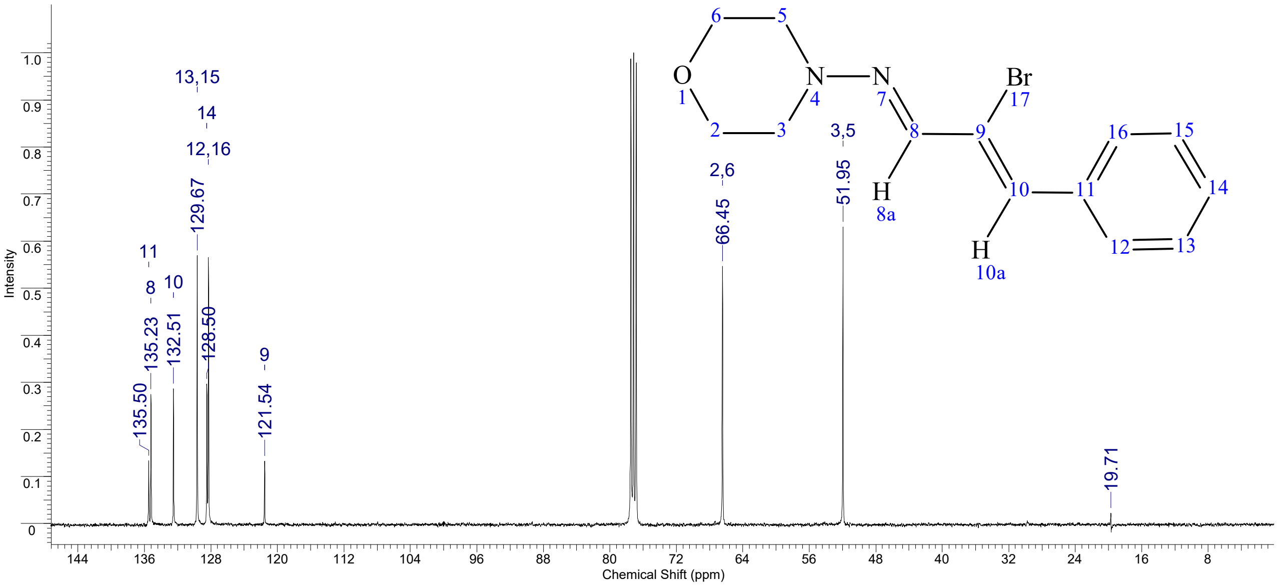

Fig. S2. ^{13}C (100.53 MHz, $CDCl_3$) NMR Spectra of **8**

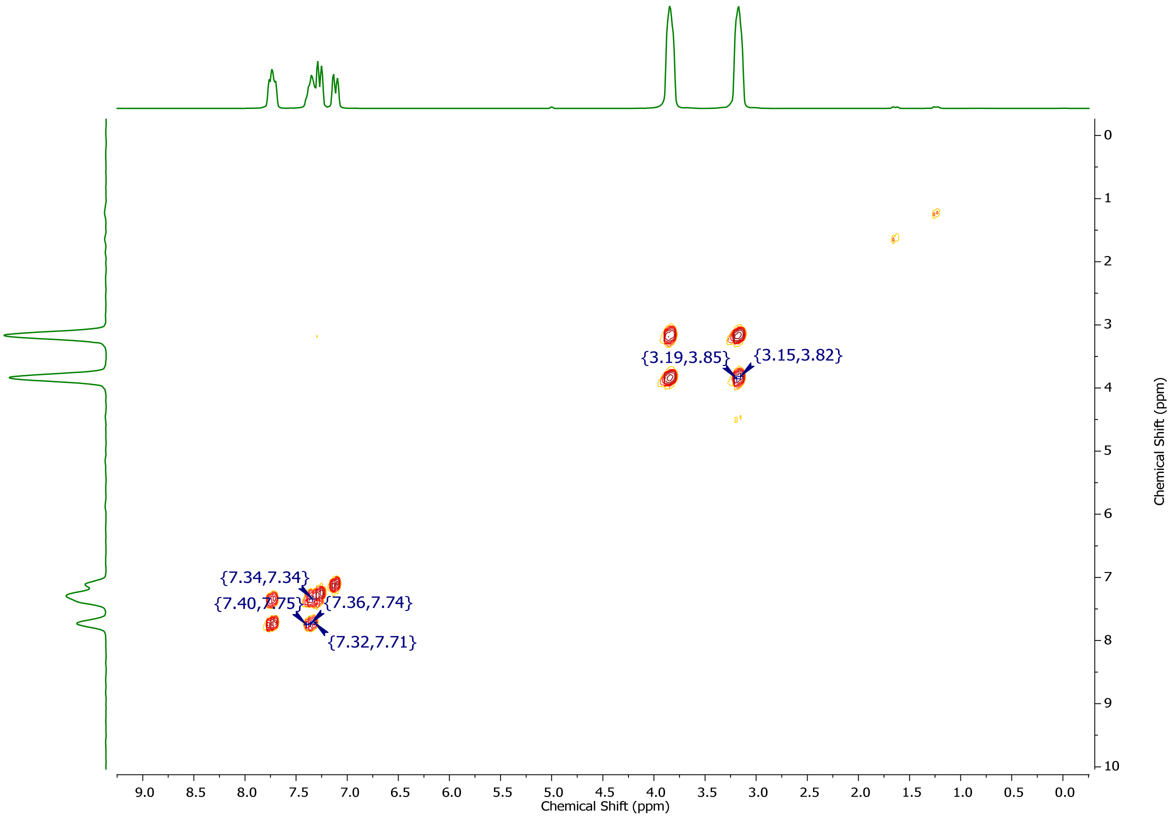

Fig. S3. COSY of **8** in_ $CDCl_3$

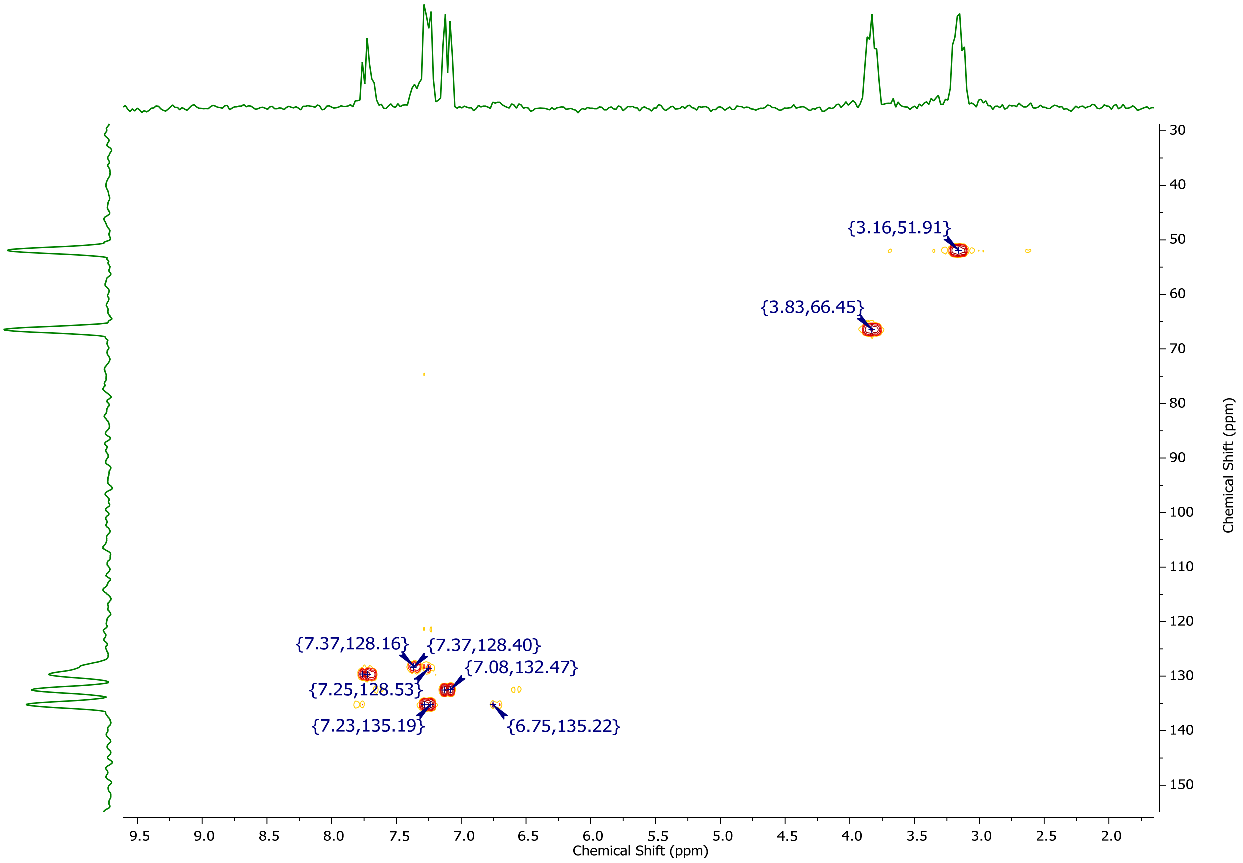

Fig. S4. HMQC of **8** in_ $CDCl_3$

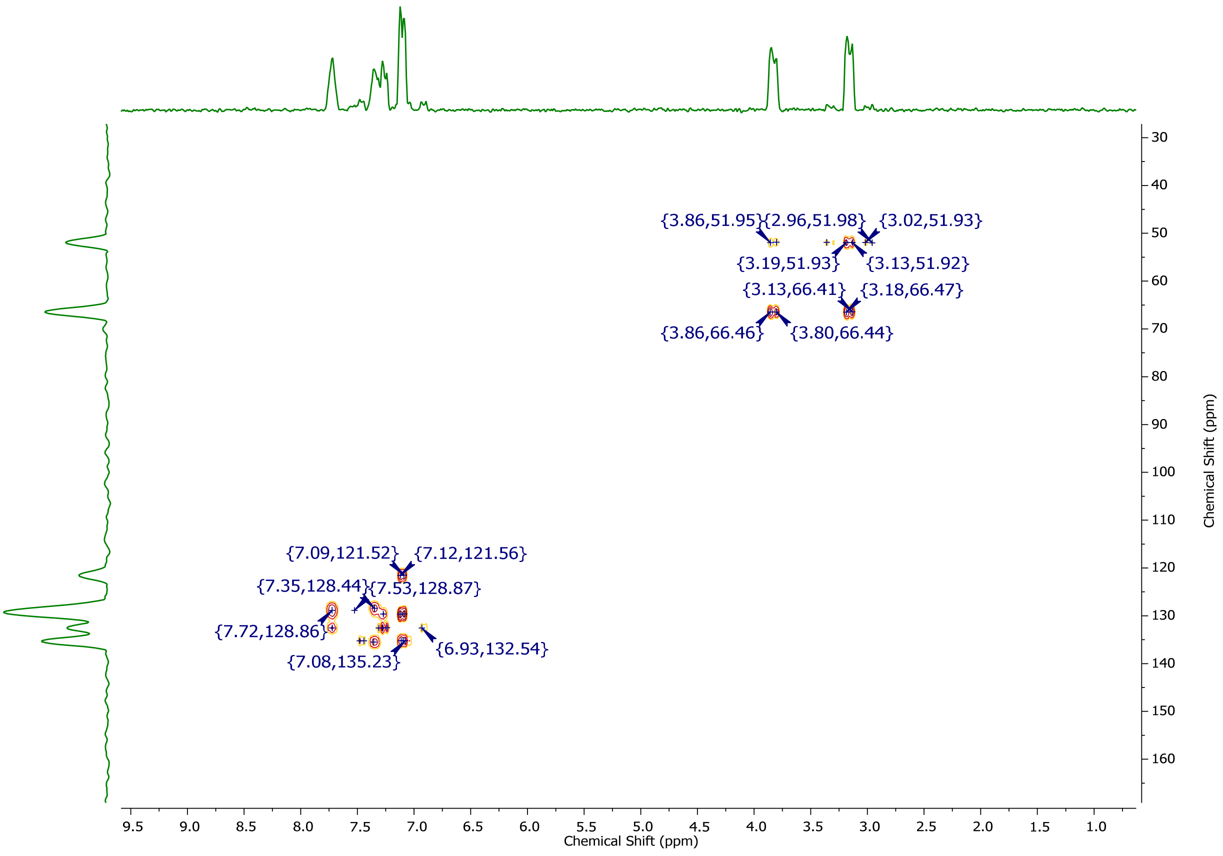

Fig. S5. HMBC of **8** in_ CDCl3

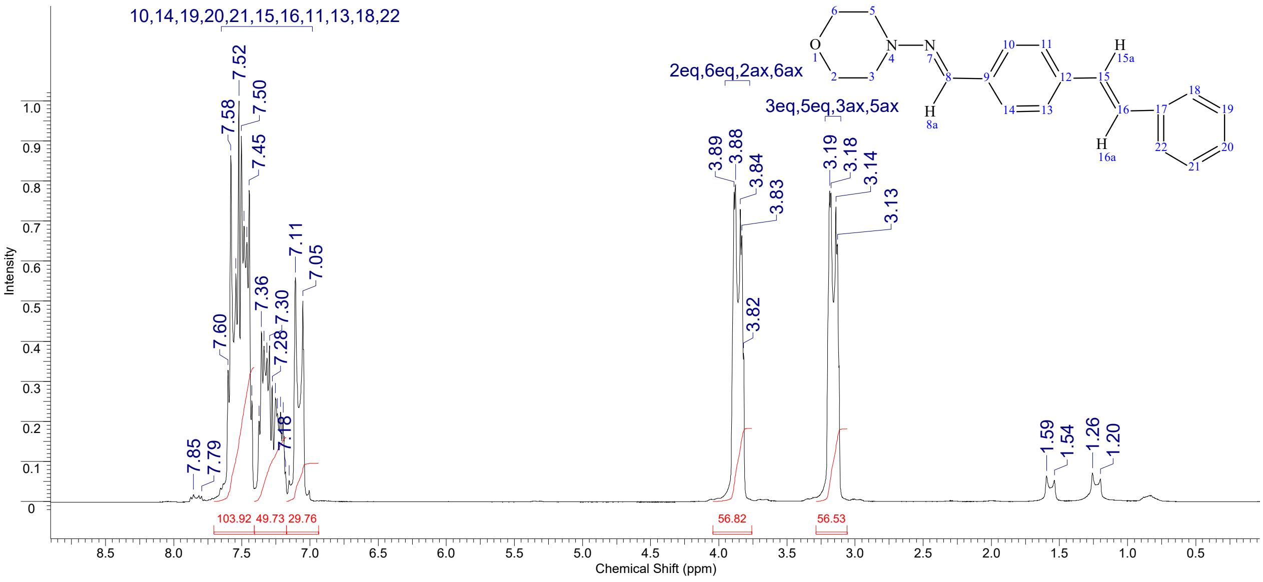

Fig. S6. ^{1}H (399.78 MHz, CDCl3) NMR Spectra of **9**

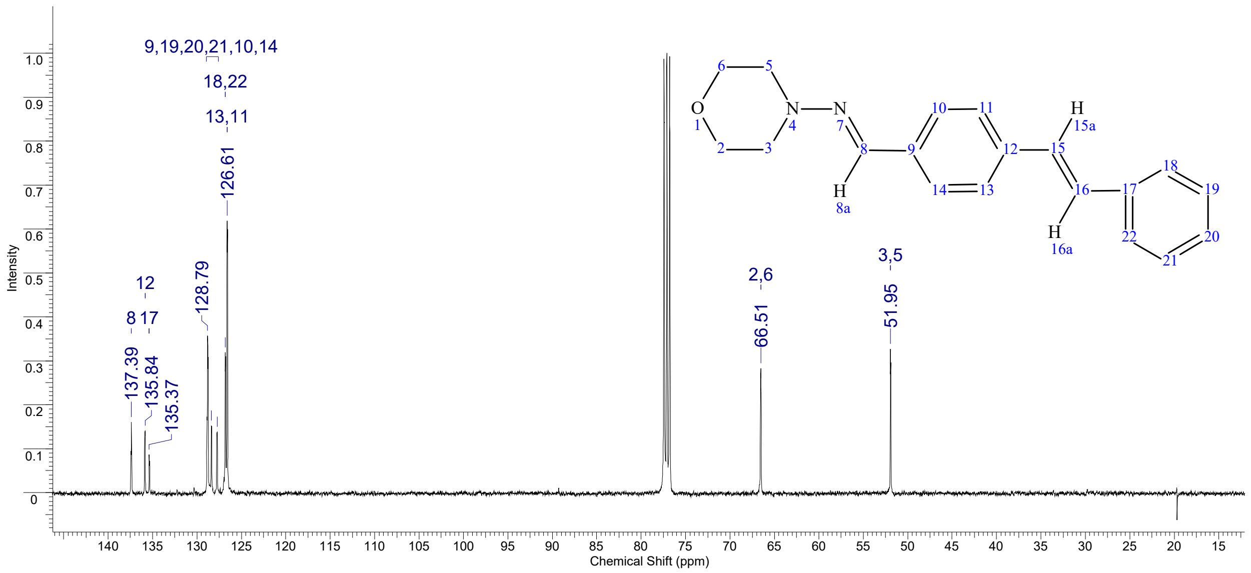

Fig. S7. ^{13}C (100.53 MHz, $CDCl_3$) NMR Spectra of **9**

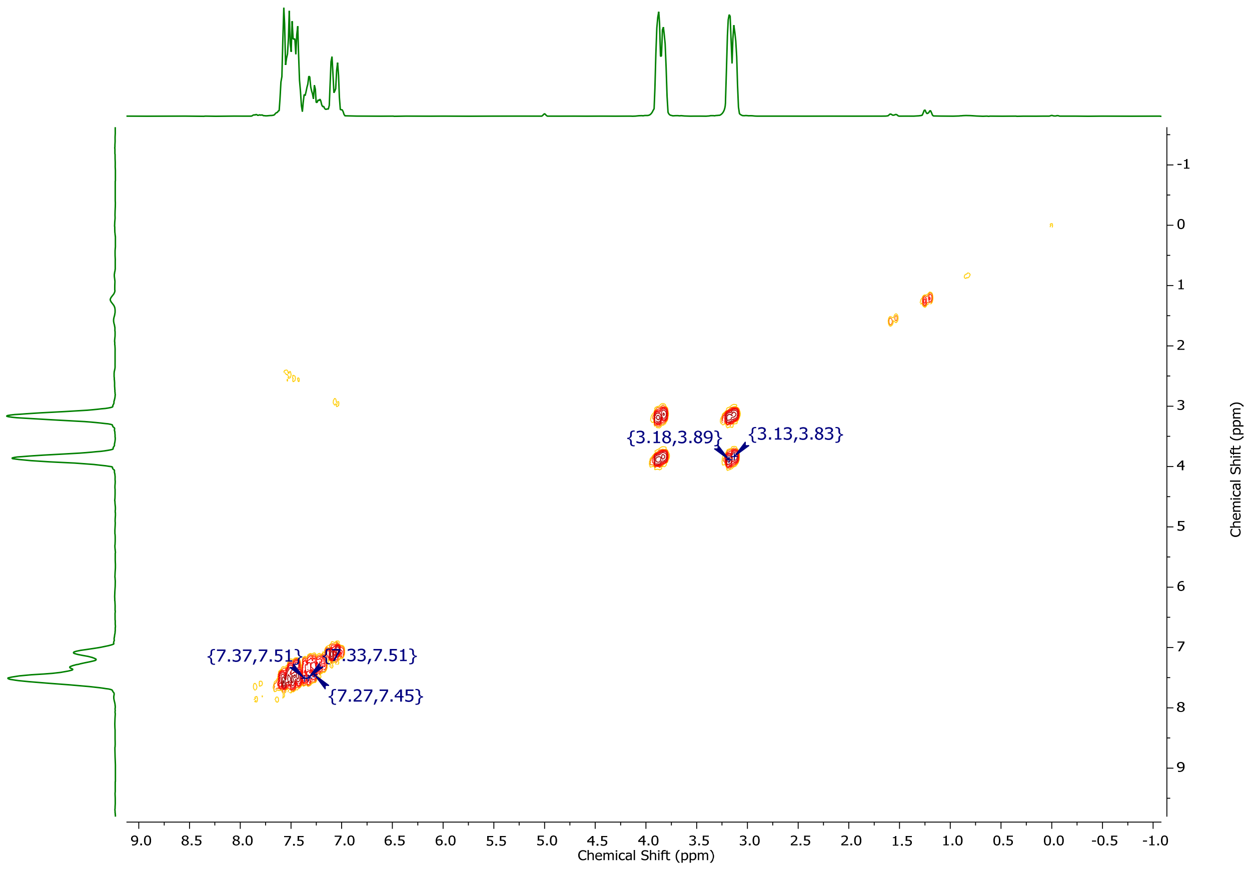

Fig. S8. COSY of **9** in_ $CDCl_3$

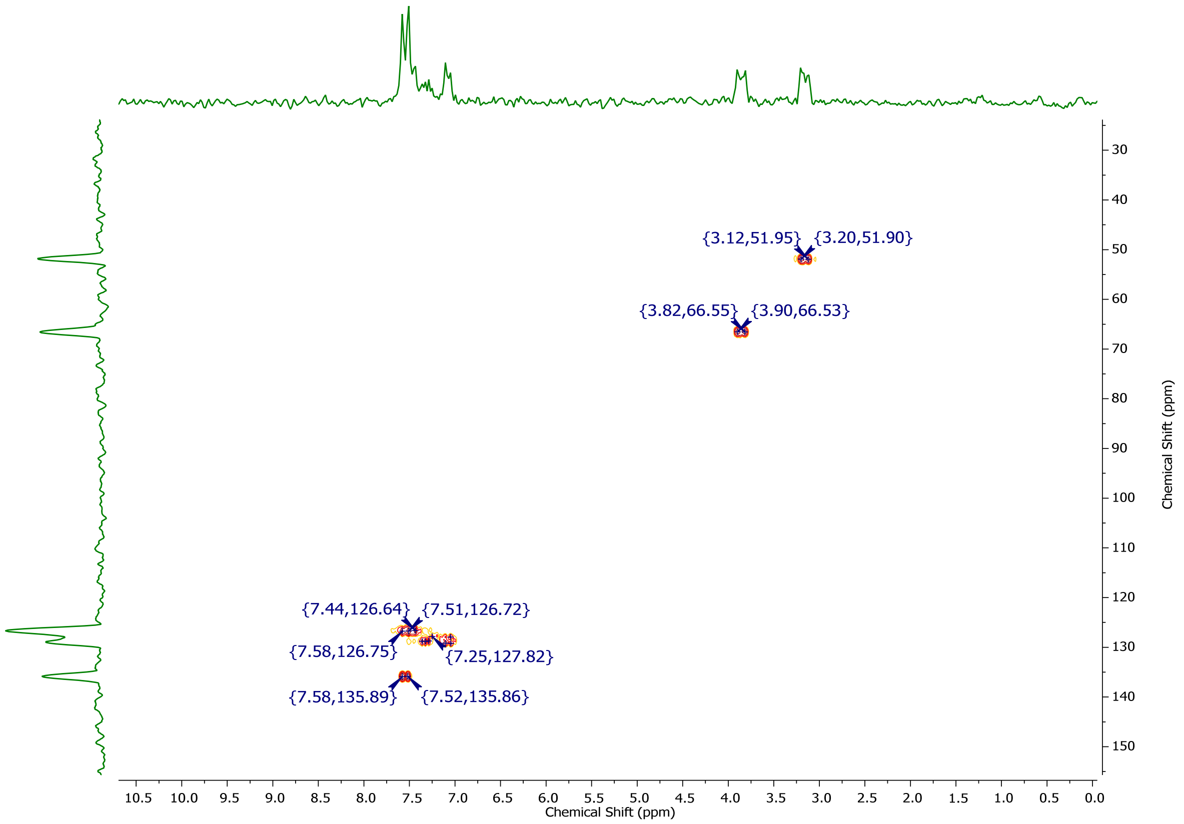

Fig. S9. HMQC of **9** in_ CDCl₃

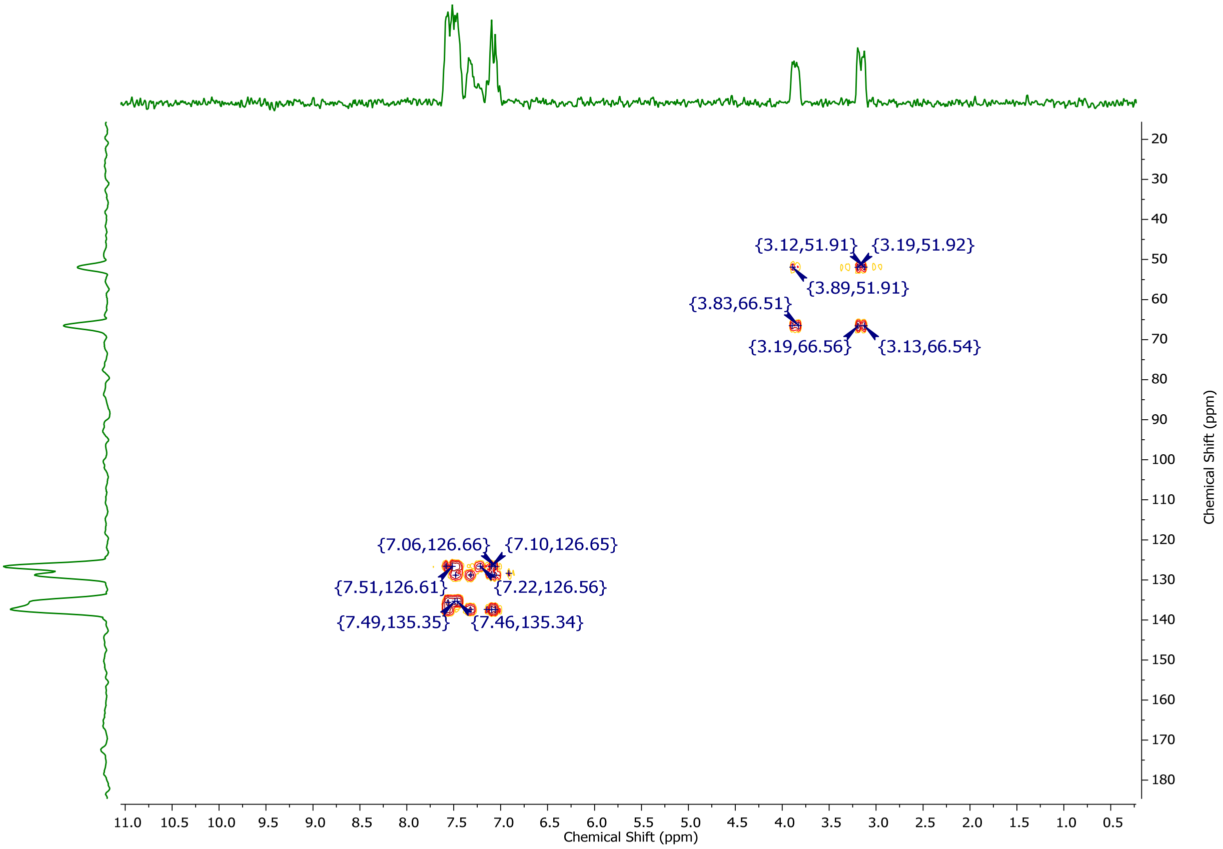

Fig. S10. HMBC of **9** in_ $CDCl_3$

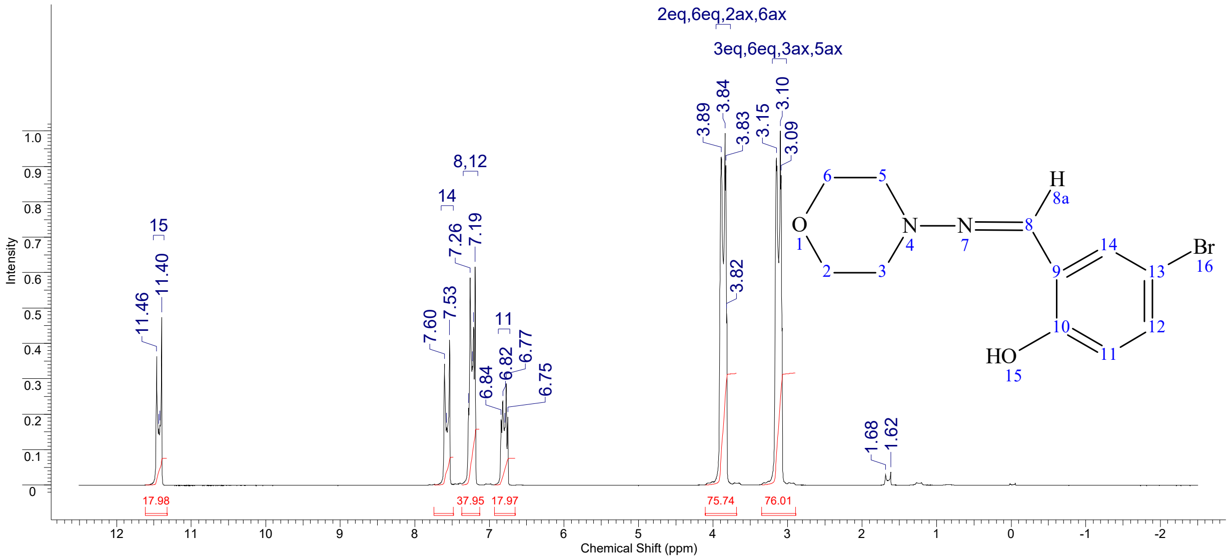

Fig. S11. ^{1}H (399.78 MHz, $CDCl_3$) NMR Spectra of **10**

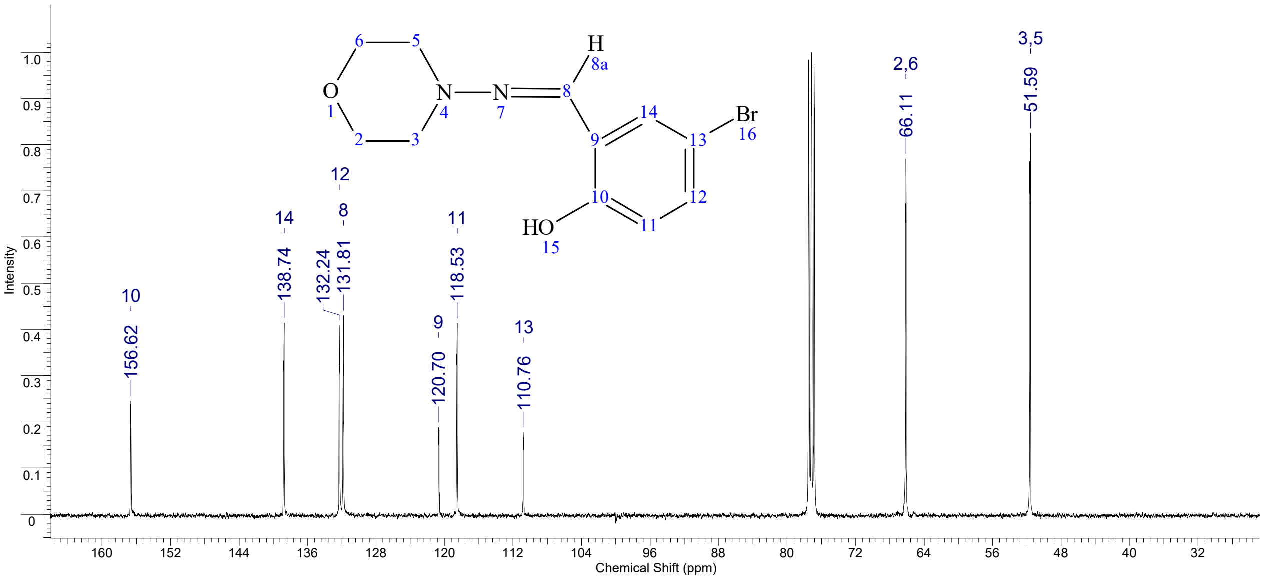

Fig. S12. ^{13}C (100.53 MHz, $CDCl_3$) NMR Spectra of **10**

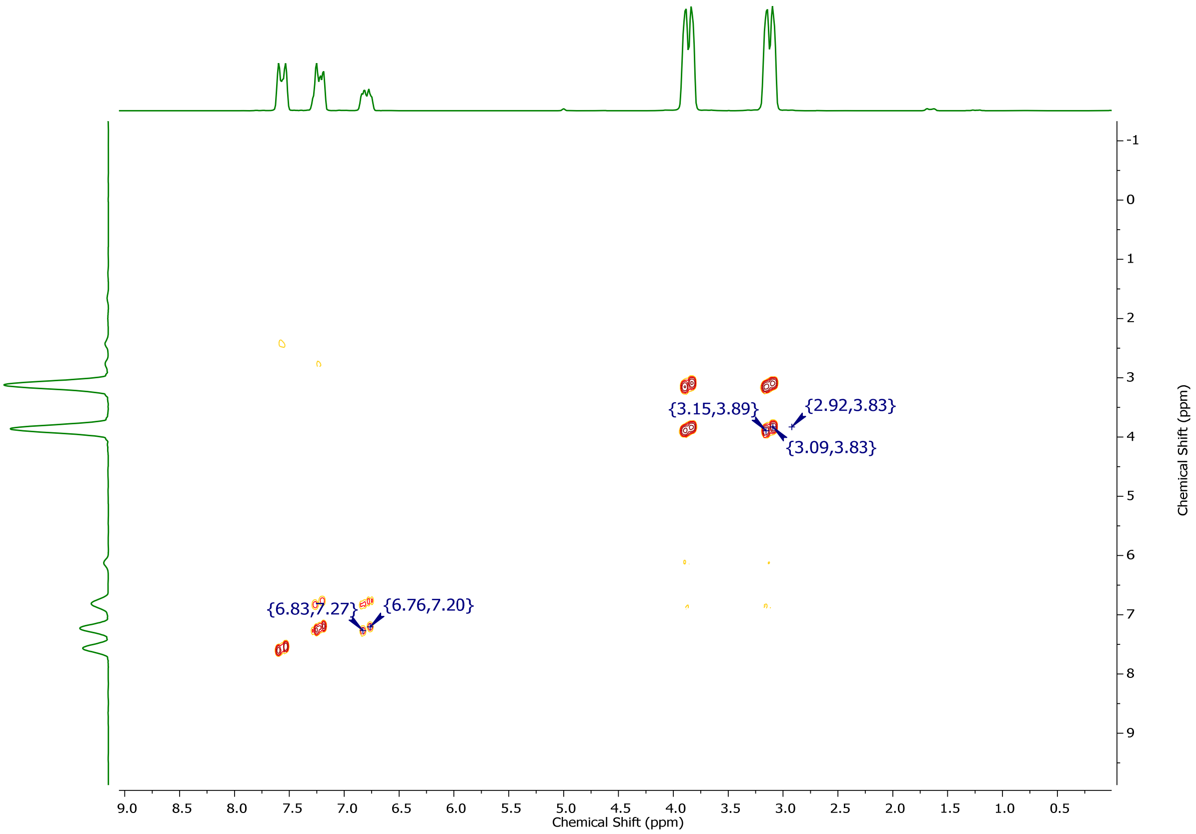

Fig. S13. COSY of **10** in_ $CDCl_3$

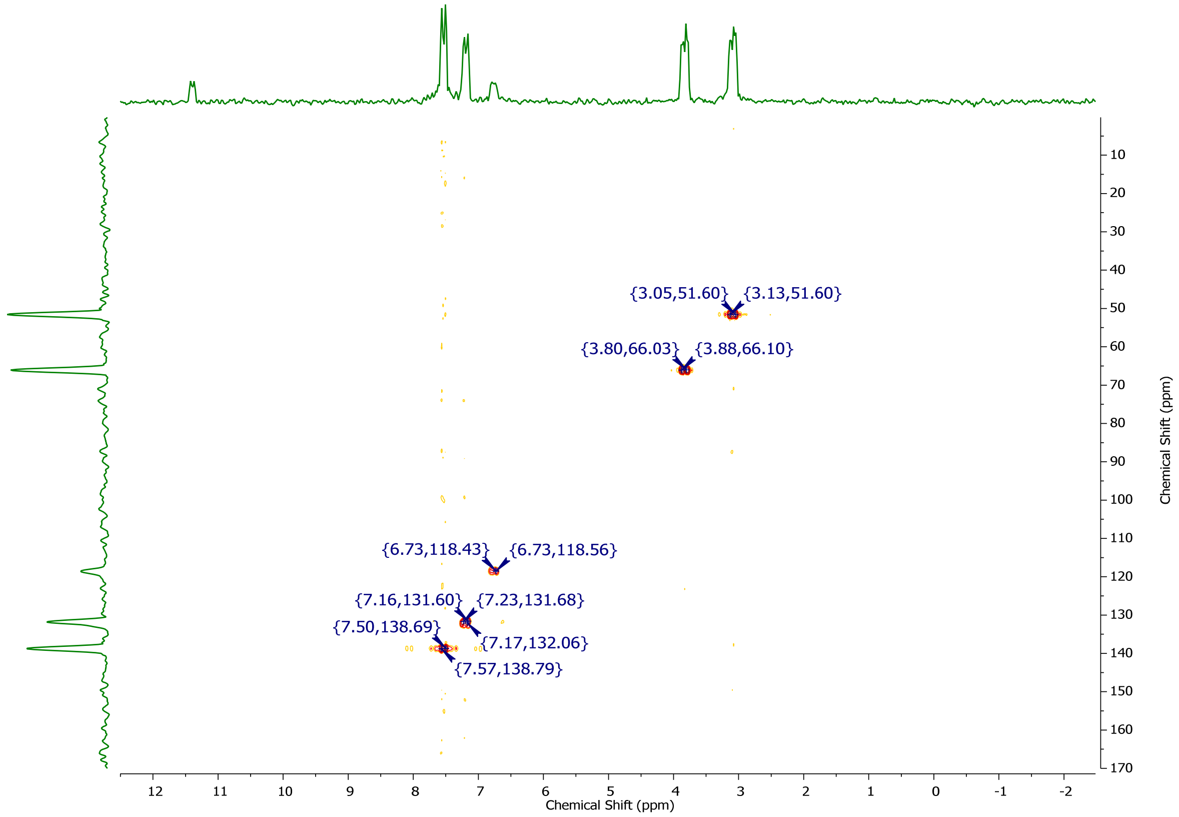

Fig. S14. HMQC of **10** in_ $CDCl_3$

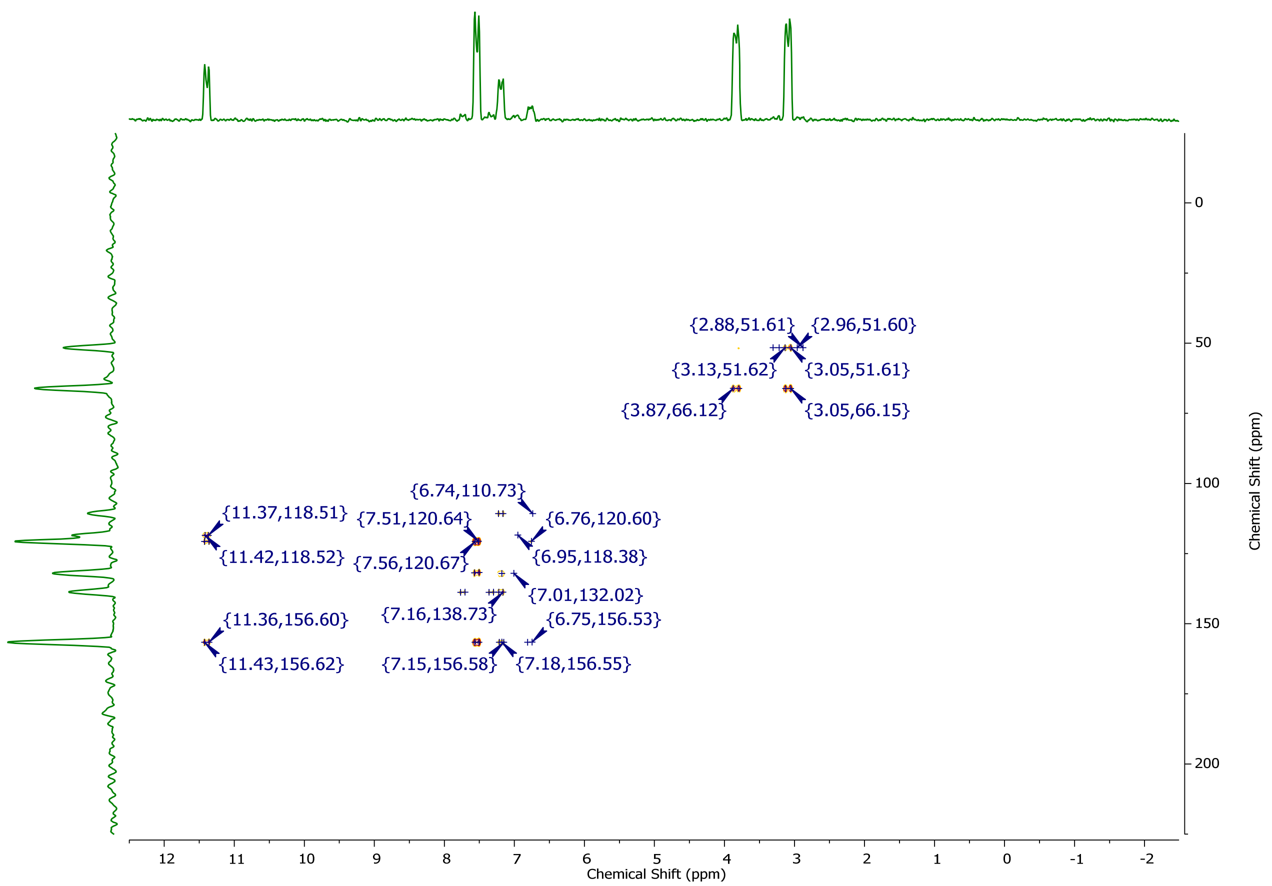

Fig. S15. HMBC of **10** in_ CDCl3

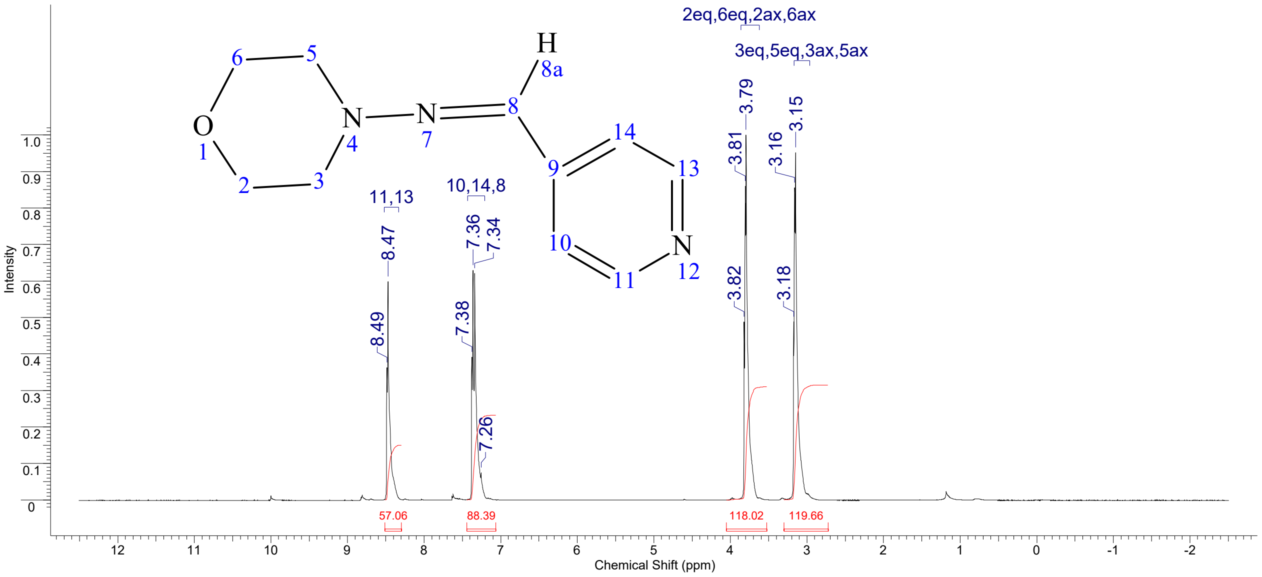

Fig. S16. ^{1}H (399.78 MHz, CDCl3) NMR Spectra of **11**

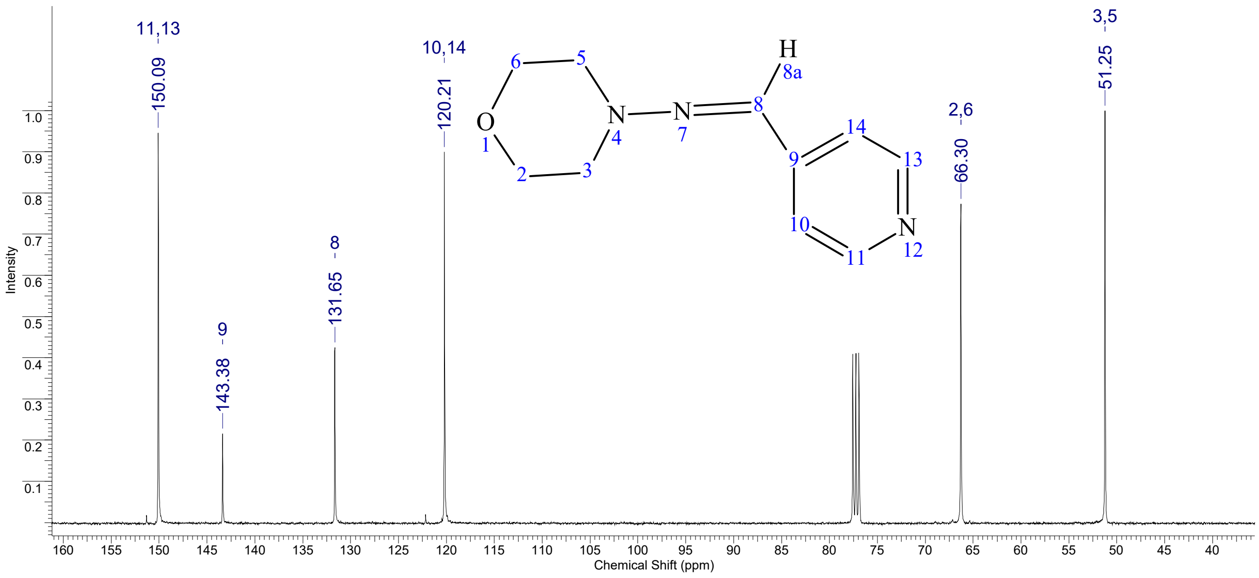

Fig. S17. and ^{13}C (100.53 MHz, $CDCl_3$) NMR Spectra of **11**

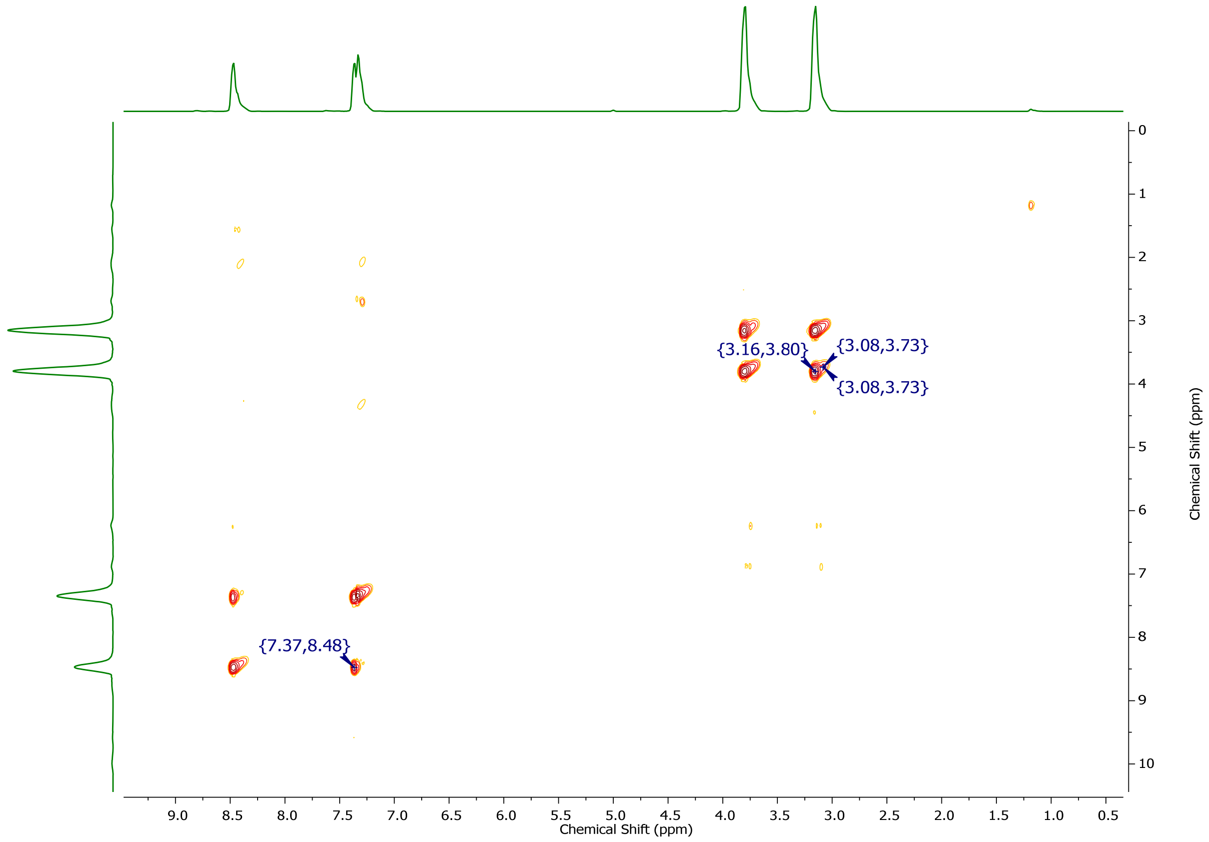

Fig. S18. COSY of **11** in_ $CDCl_3$

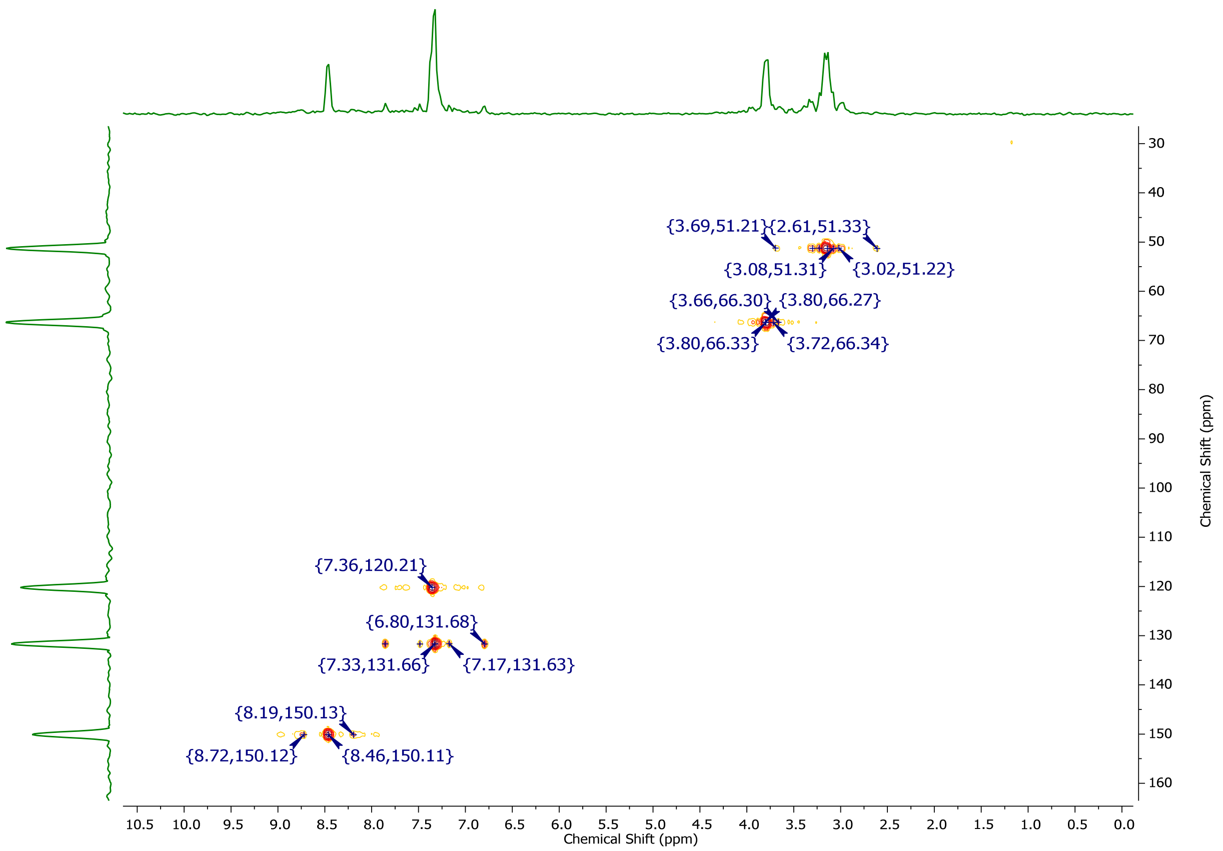

Fig. S19. HMQC of **11** in_ $CDCl_3$

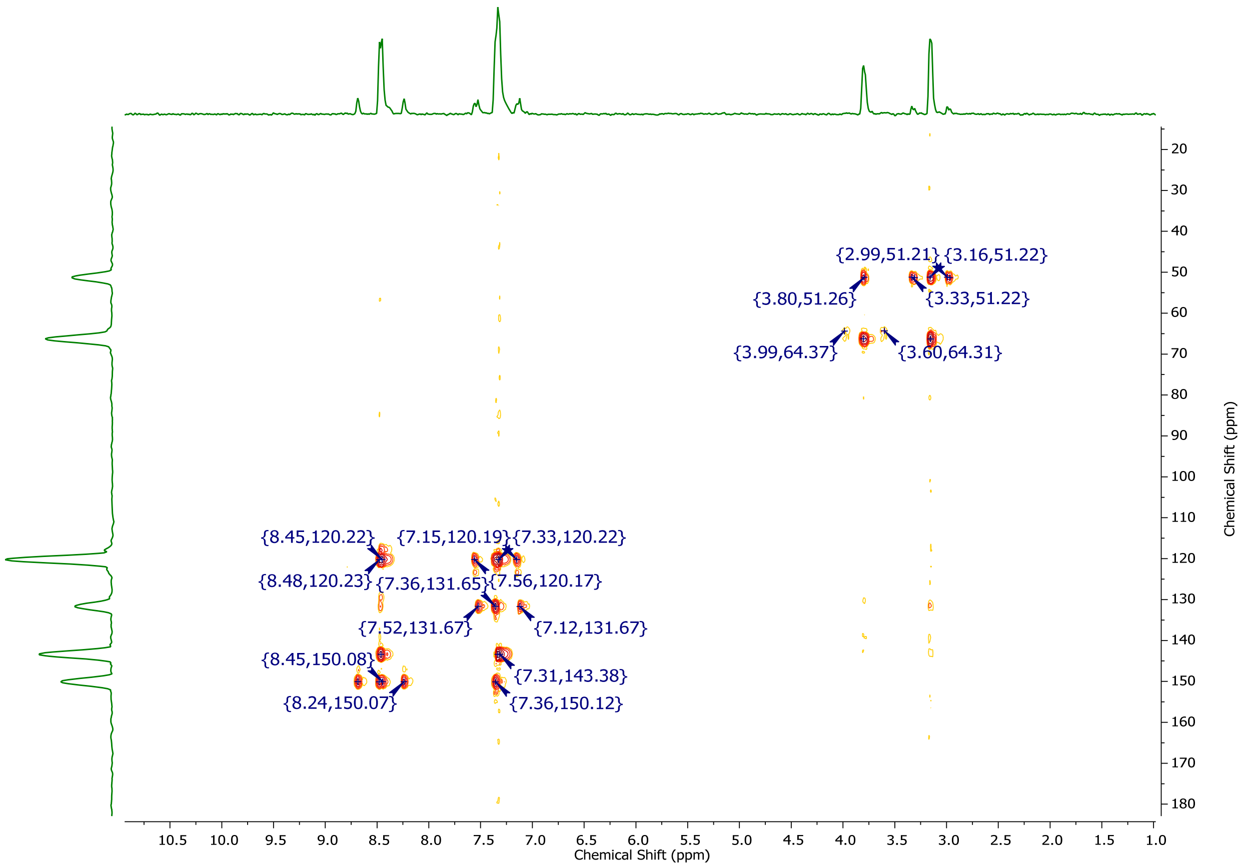

Fig. S20. HMBC of **11** in_ $CDCl_3$

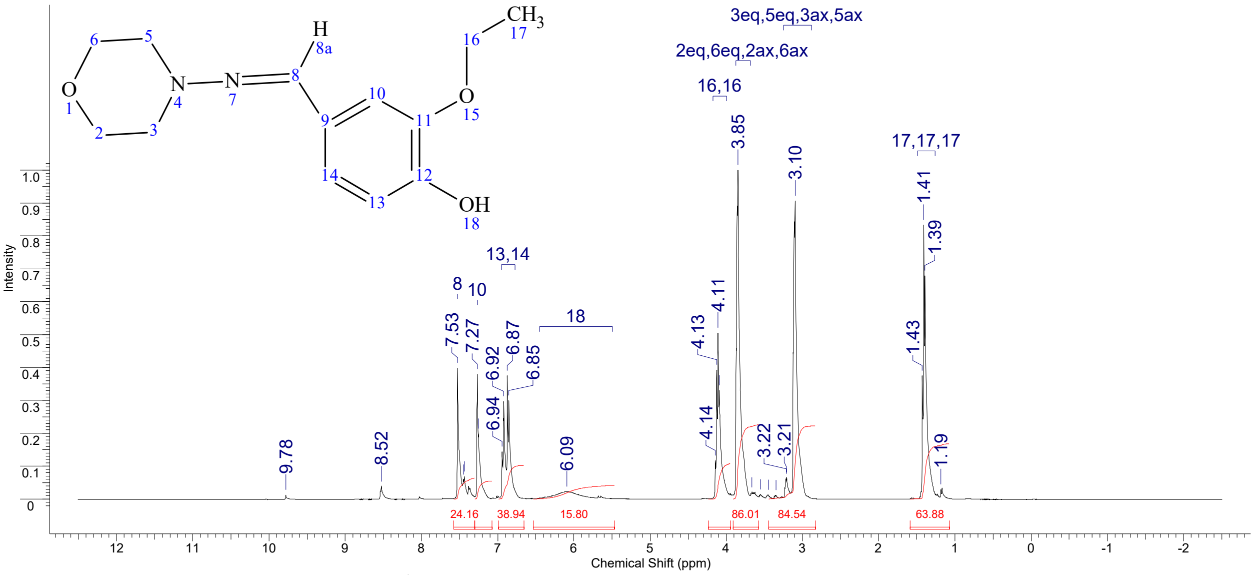

Fig. S21. ^{1}H (399.78 MHz, $CDCl_3$) NMR Spectra of **12**

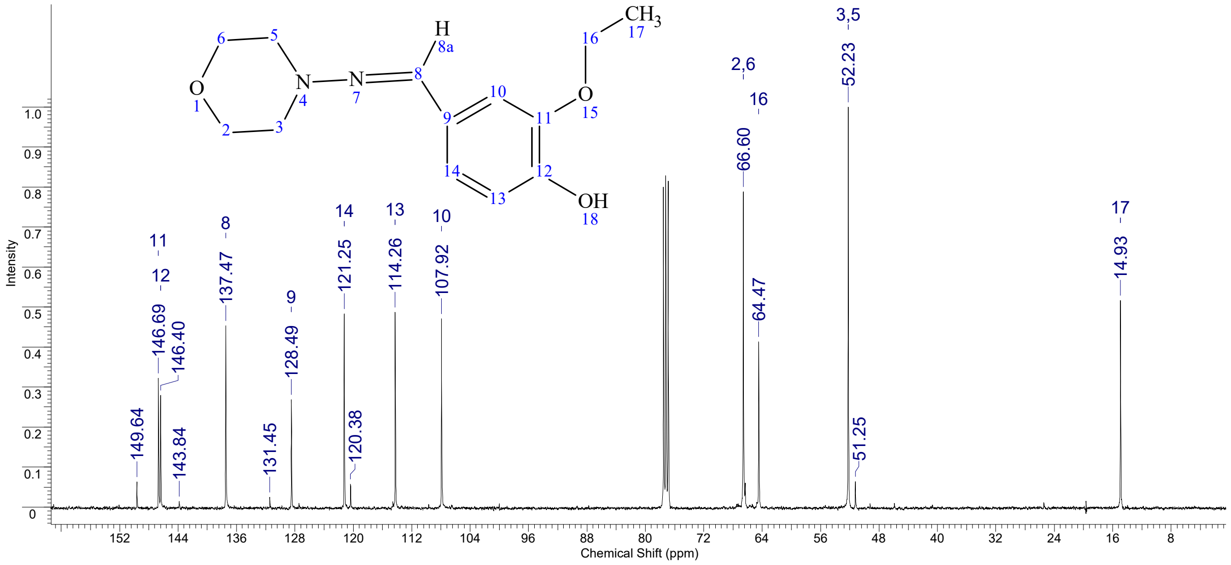

Fig. S22. ^{13}C (100.53 MHz, $CDCl_3$) NMR Spectra of **12**

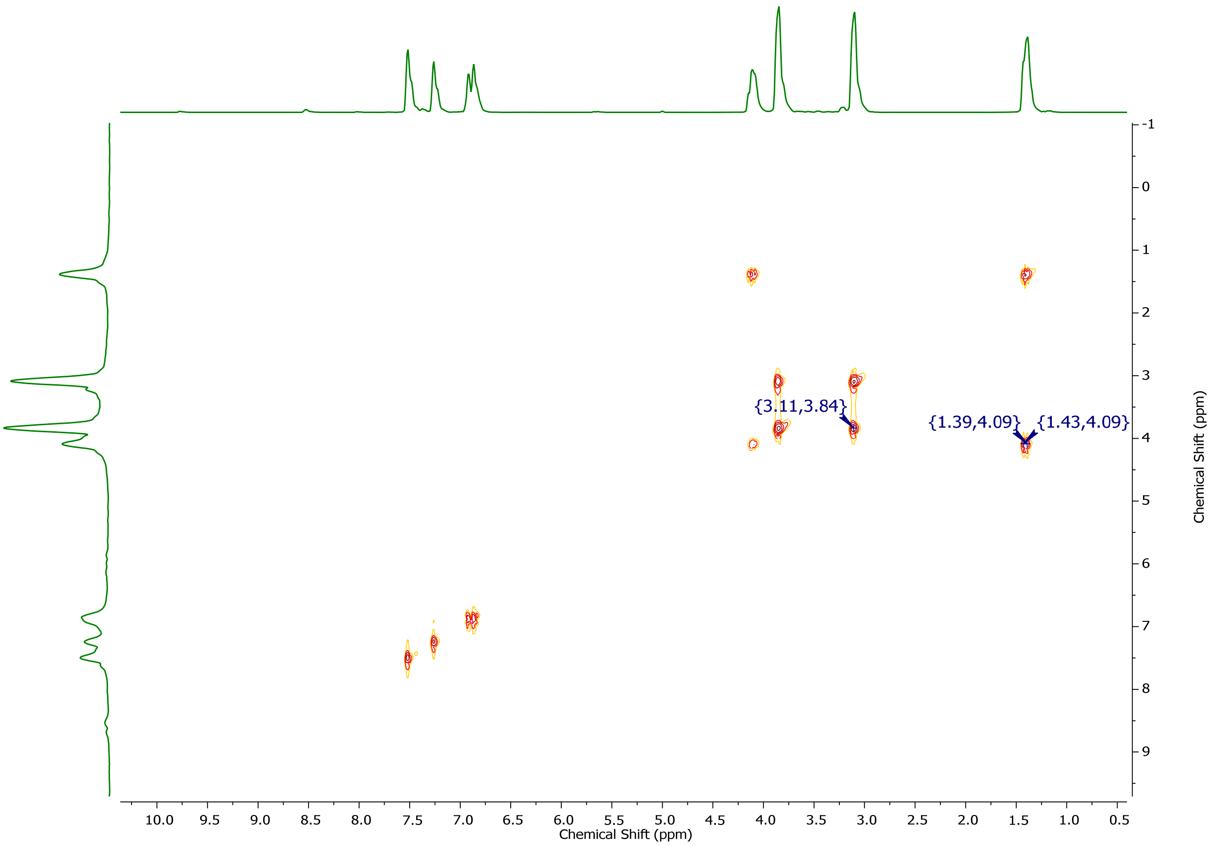

Fig. S23. COSY of **12** in_ $CDCl_3$

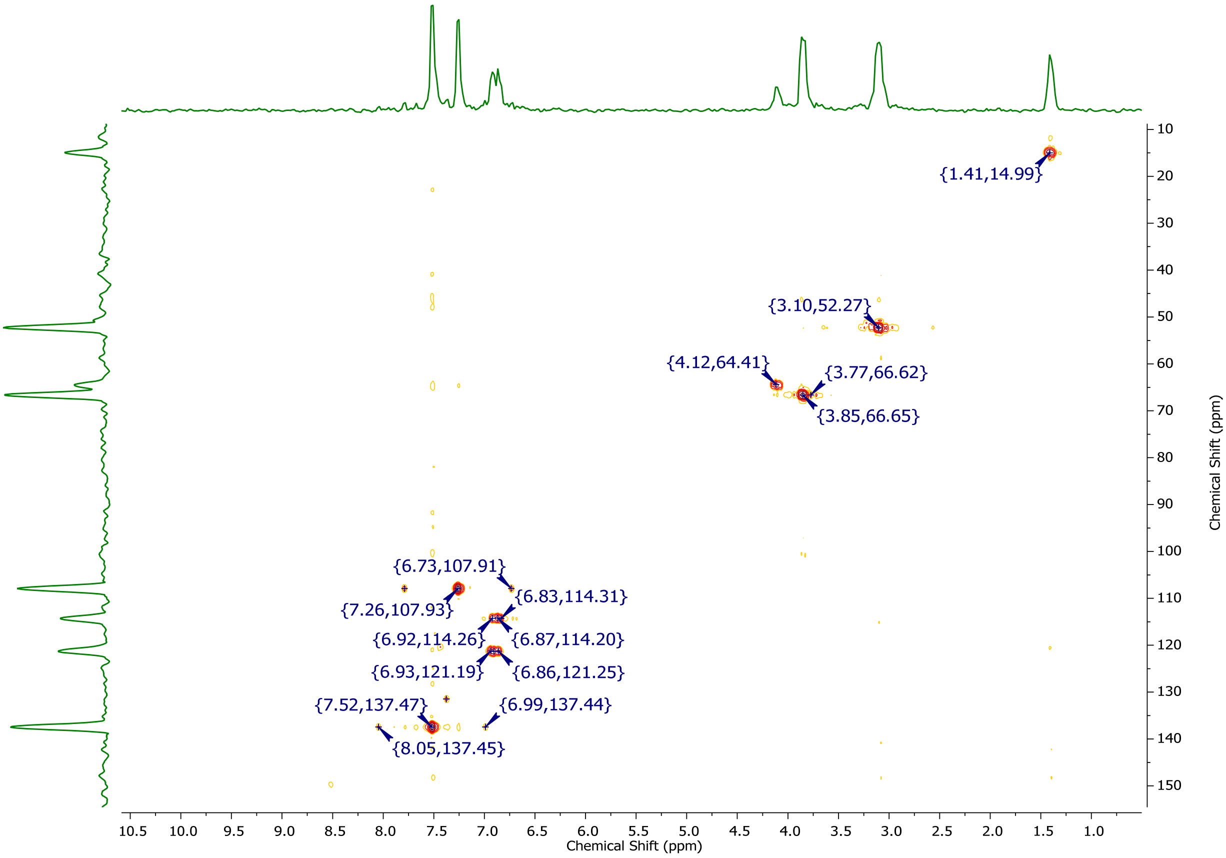

Fig. S24. HMQC of **12** in_ $CDCl_3$

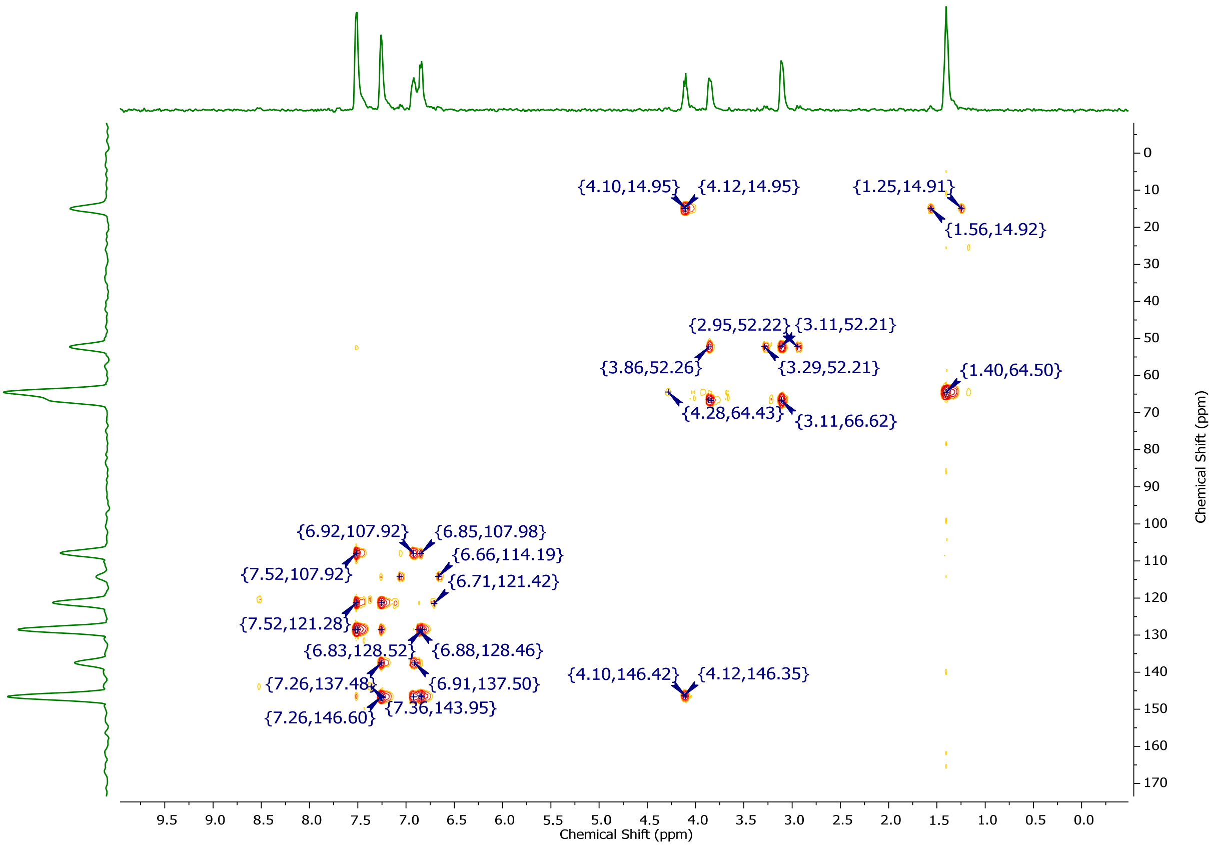

Fig. S25. HMBC of **12** in_ $CDCl_3$

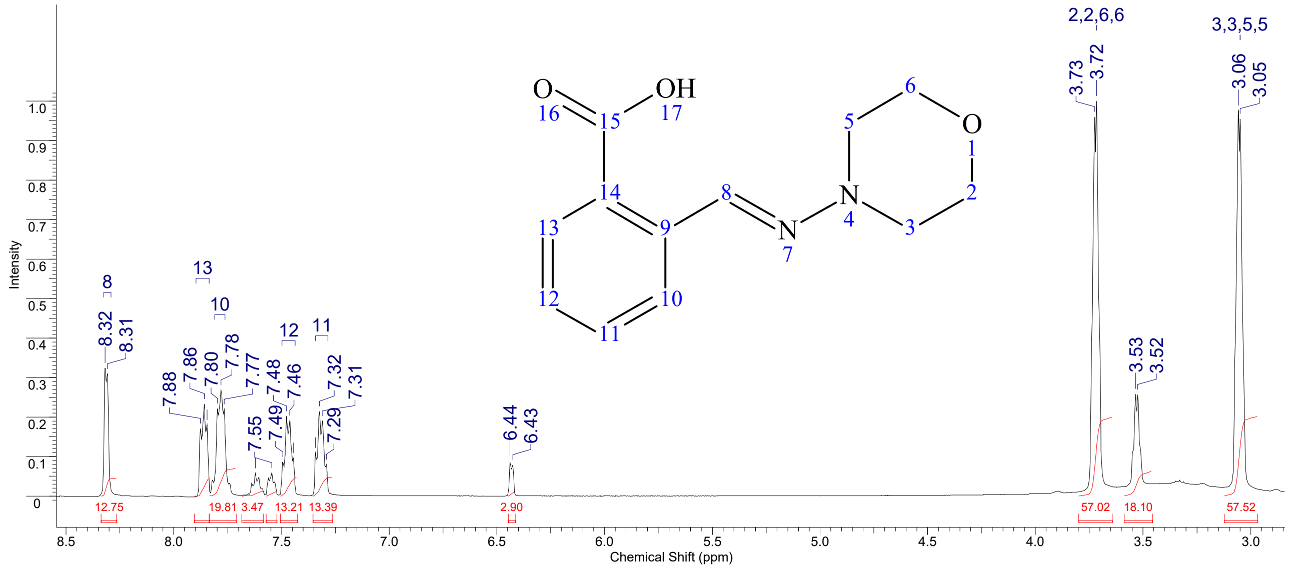

Fig. S26. ^{1}H (399.78 MHz, DMSO-d6) NMR Spectra of **13**

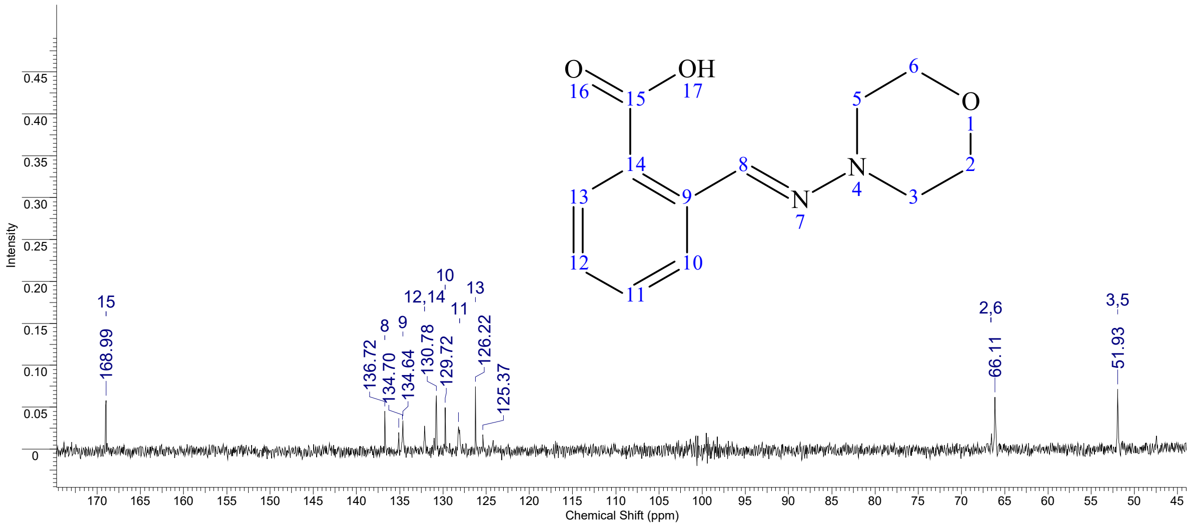

Fig. S27.^{13}C (100.53 MHz, DMSO-d$_6$) NMR Spectra of **13**

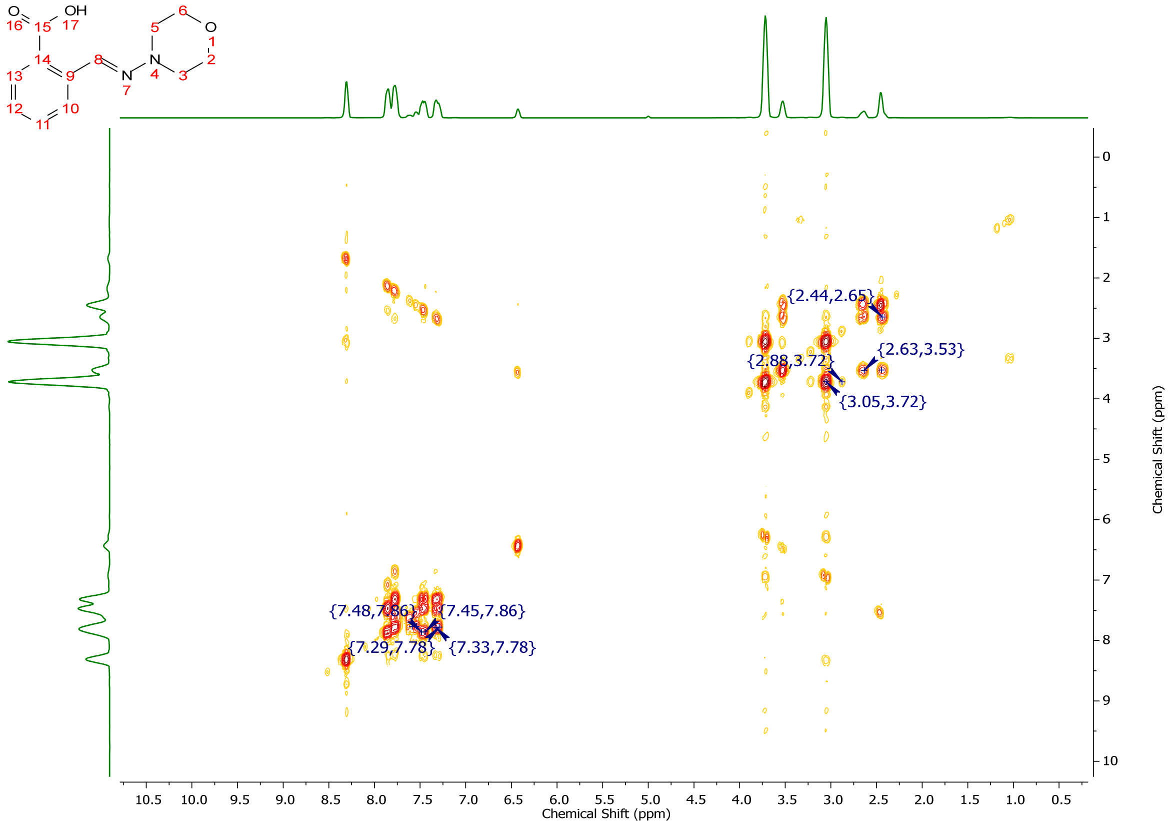

Fig. S28. COSY of **13** in_ DMSO-d$_6$

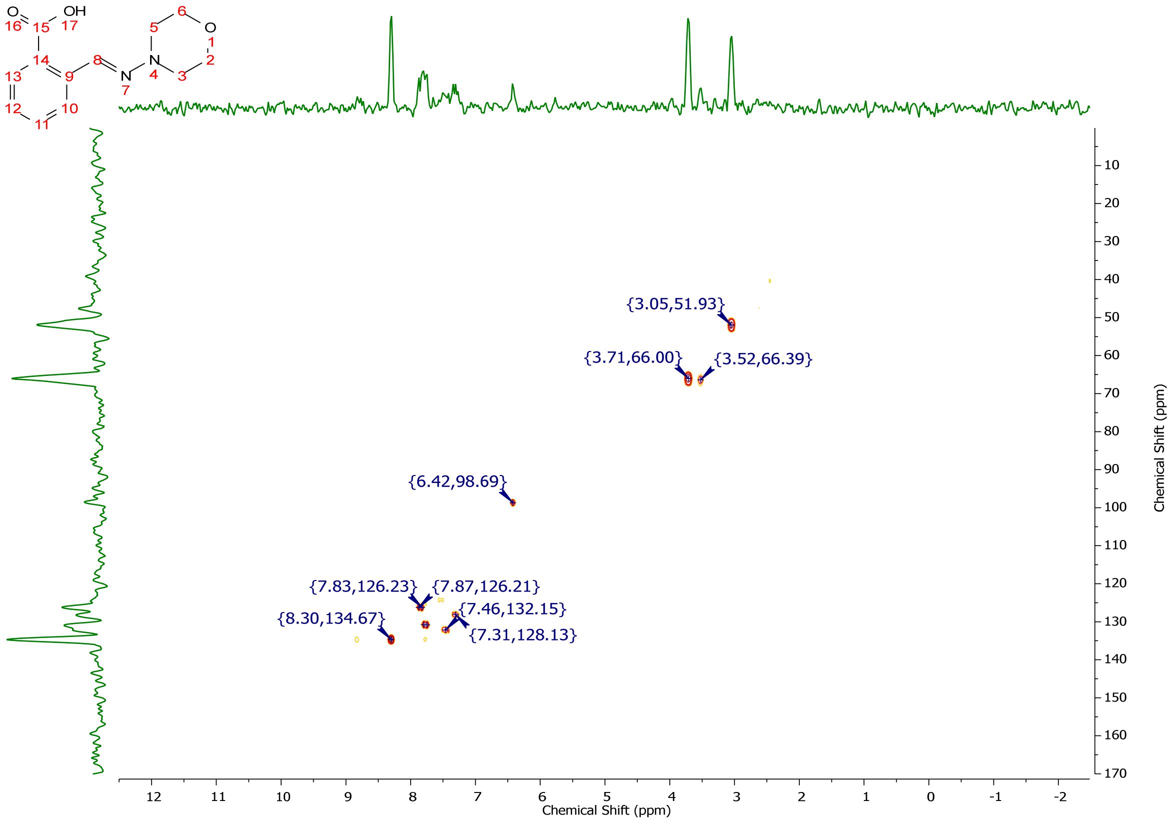

Fig. S29. HMQC of **13** in_ DMSO-d₆

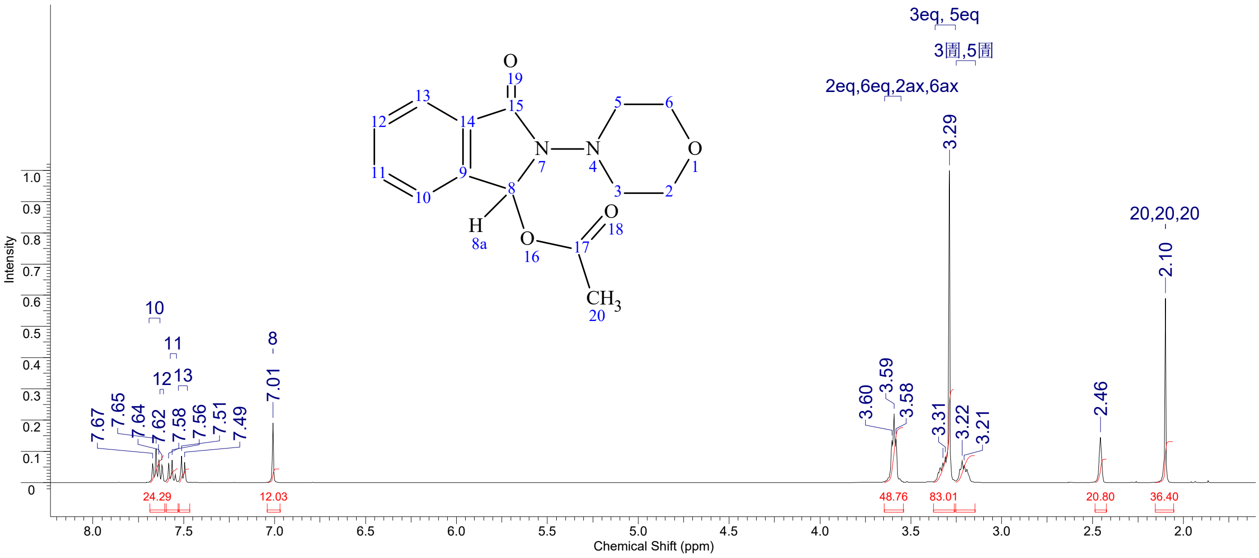

Fig. S30. ^{1}H (399.78 MHz, DMSO-d₆) NMR Spectra of **15**

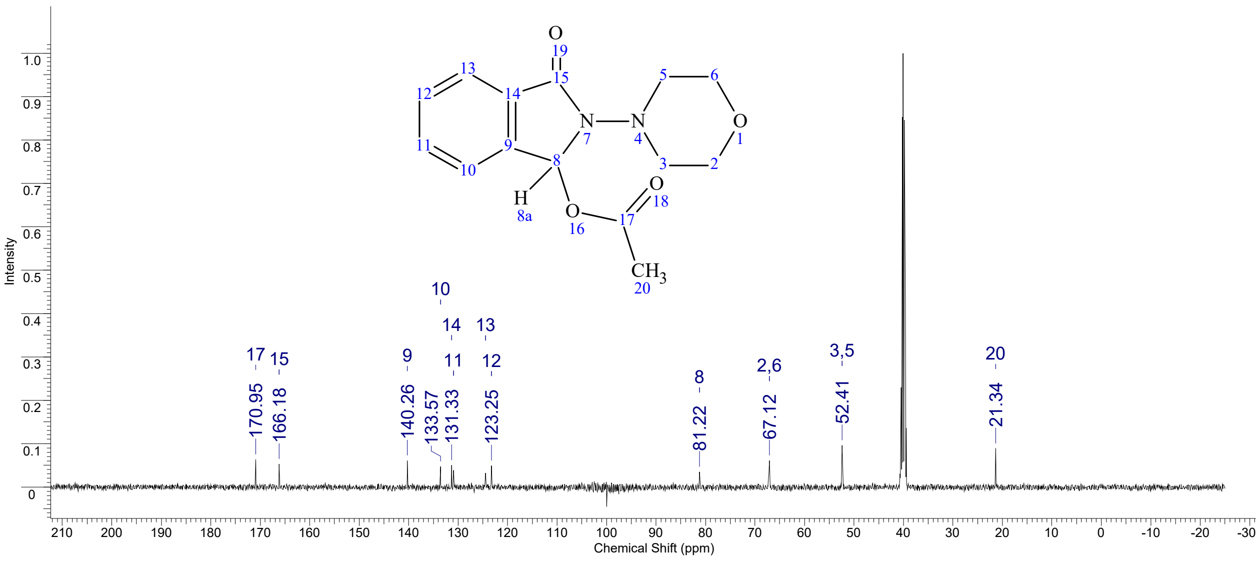

Fig. S31. ^{13}C (100.53 MHz, DMSO-d6) NMR Spectra of **15**

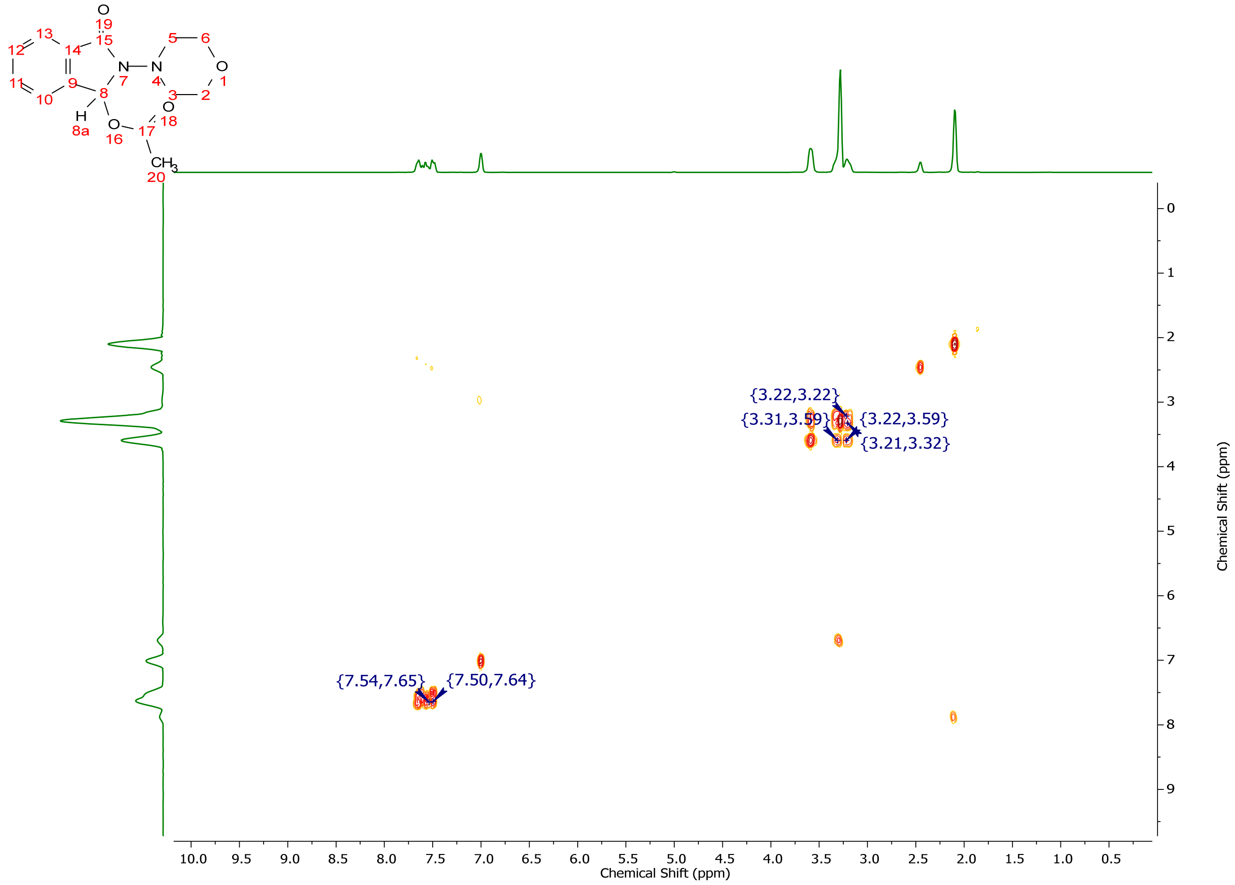

Fig. S32. COSY of **15** in_ DMSO-d6

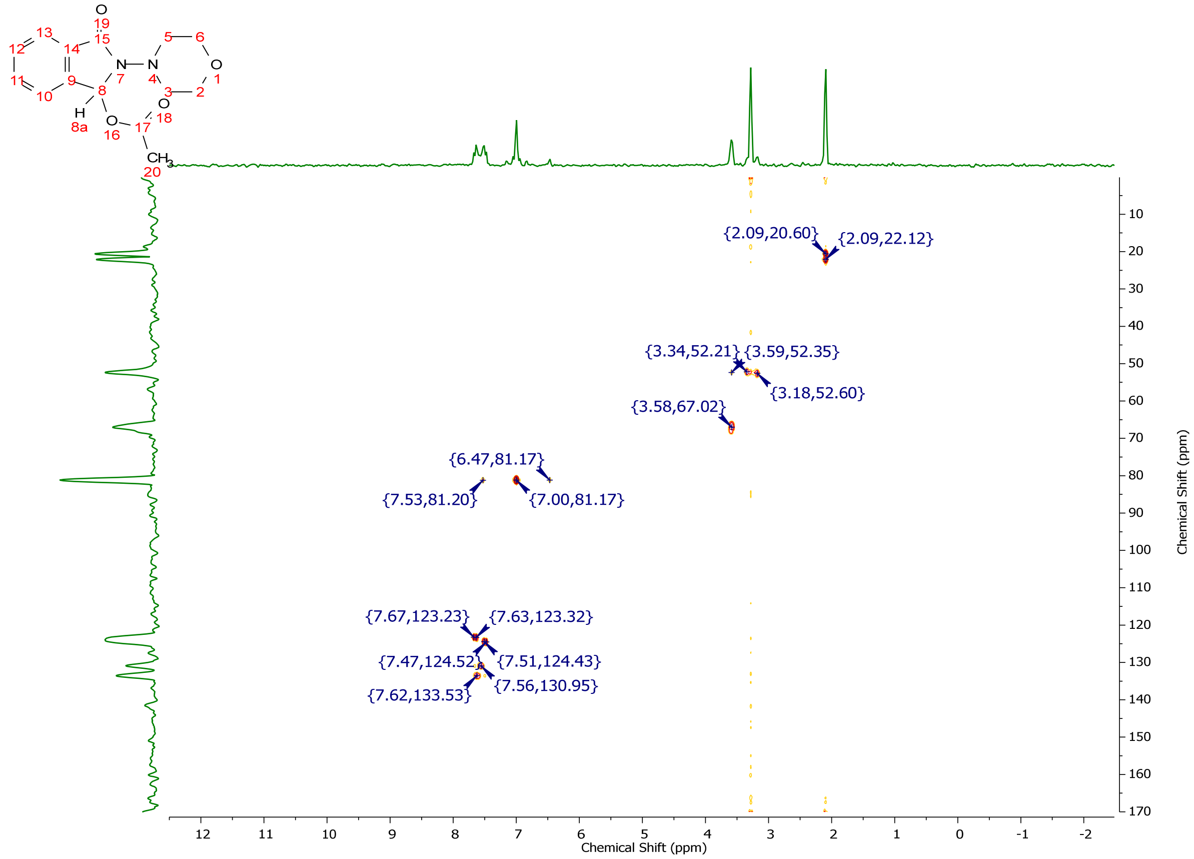

Fig. S33. HMQC of **15** in_ DMSO-d_6

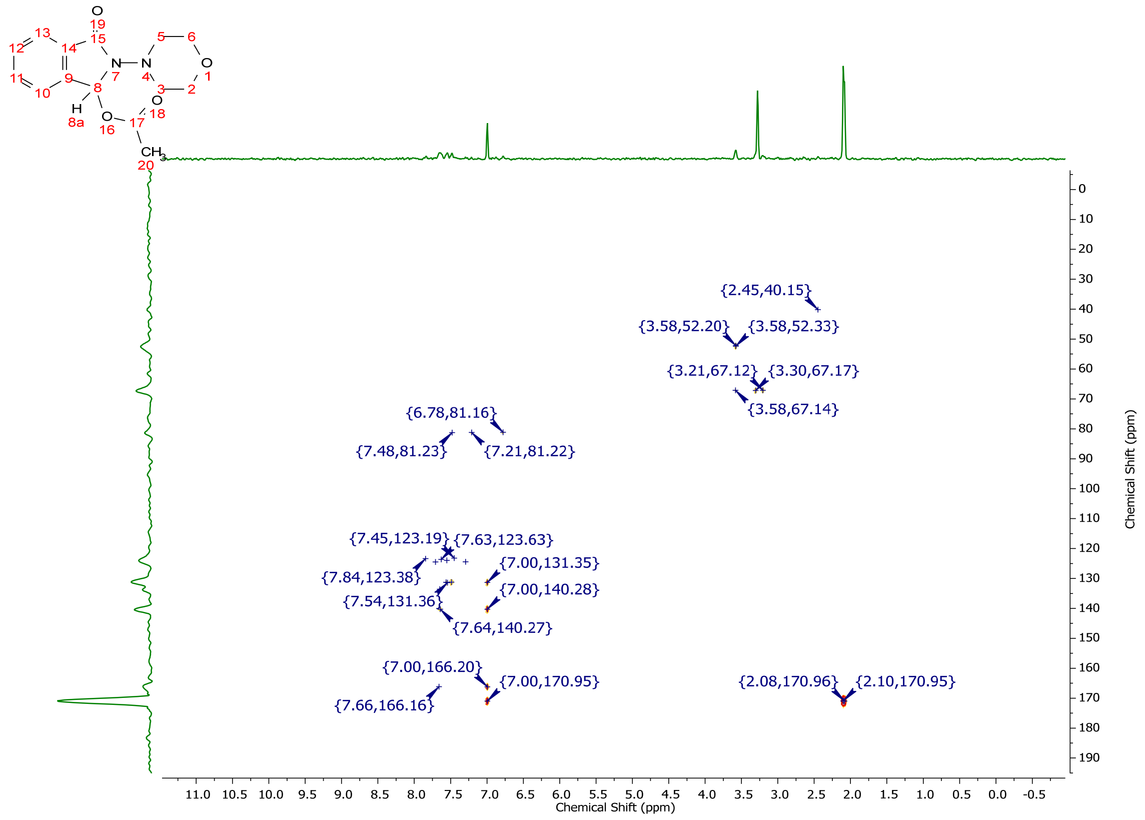

Fig. S34. HMBC of **15** in_ DMSO-d_6

Copies of MS Spectra of Products

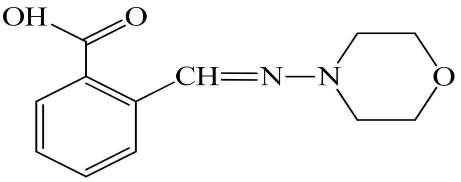

Chemical Formula: $C_{12}H_{14}N_2O_3$
Exact Mass: 234,10

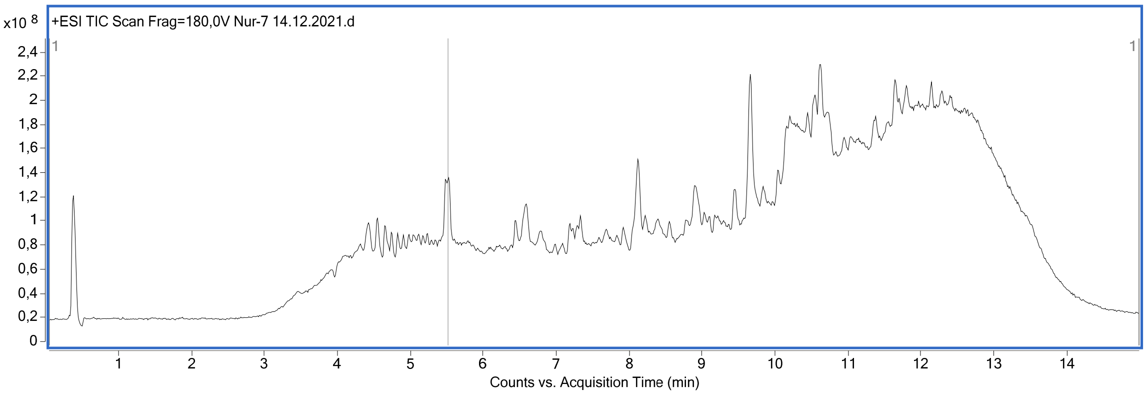

Fig. Chromatogram of compound **13**

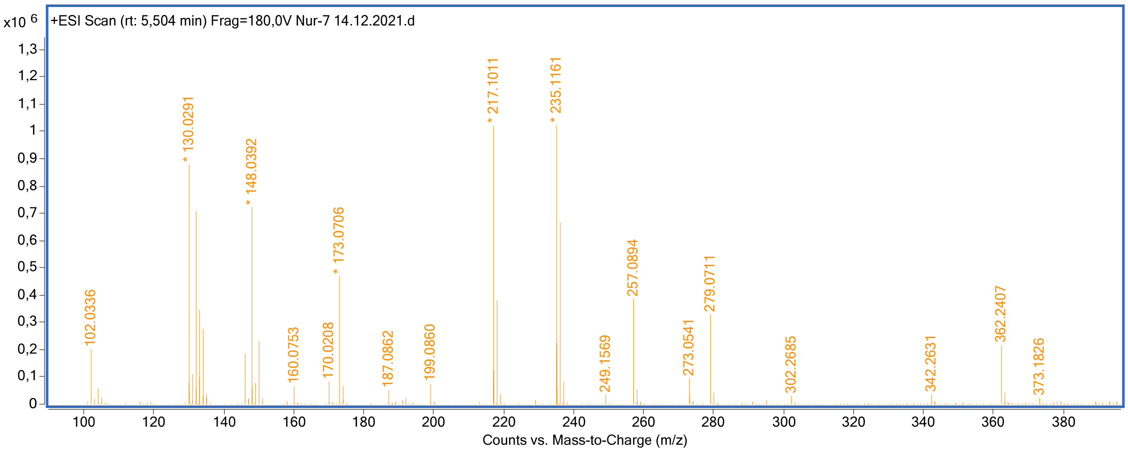

Fig. Mass spectrum of compound **13**

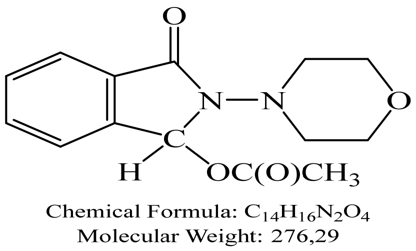

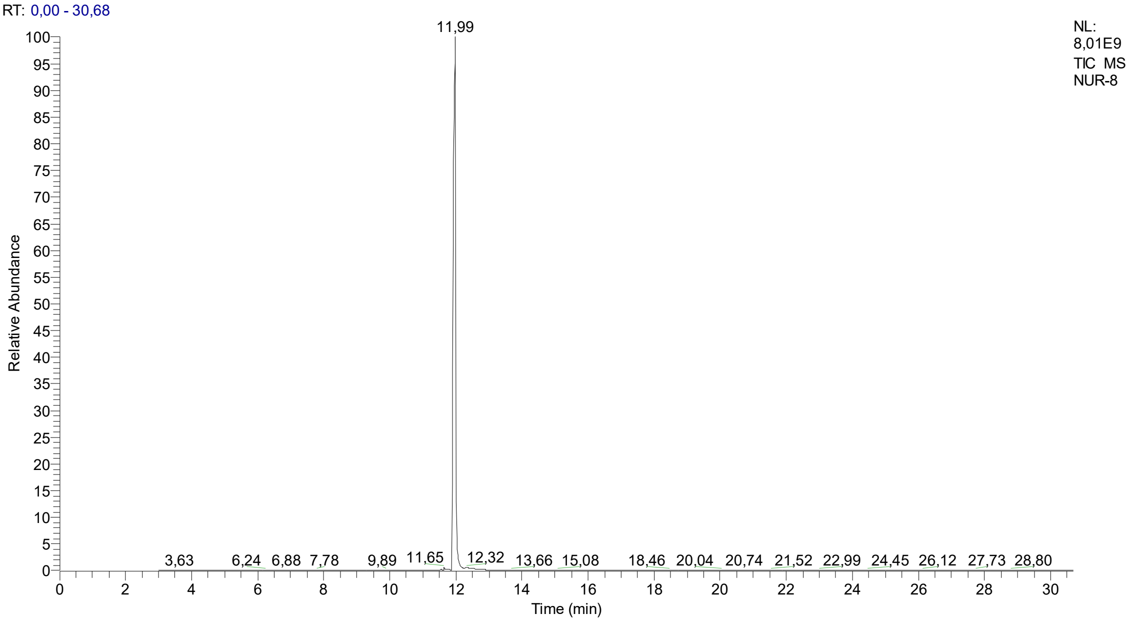

Fig. Chromatogram of compound **15**

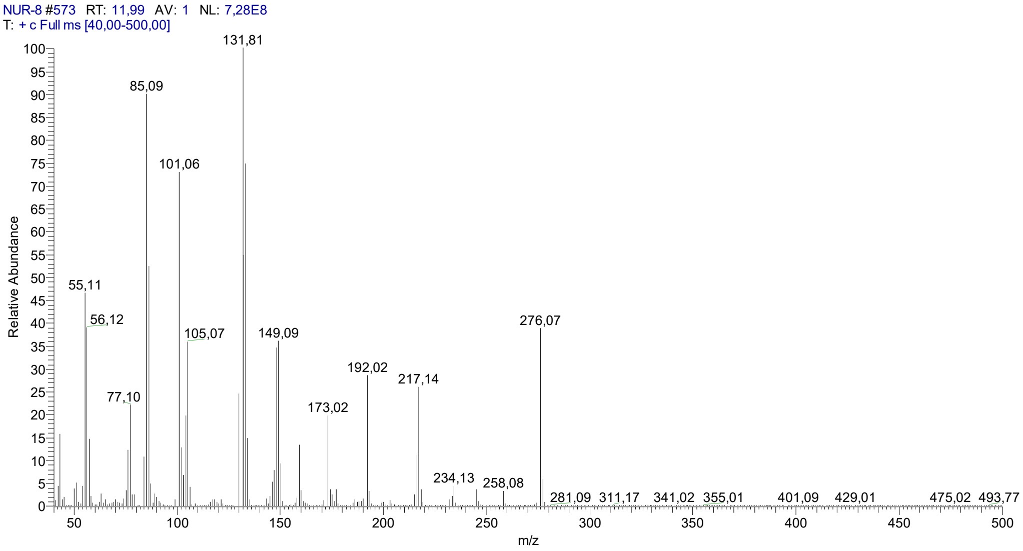

Fig. Mass spectrum of compound **15**

X-Ray Structural Study of Product 15

The X-ray diffraction study of compound **15** was performed on a Bruker APEX-II CCD diffractometer (MoK$_\alpha$, graphite monochromator, ω-scanning, 2.27°≤θ≤27.51°) at a temperature of 296 K. 21510 reflections were captured, including 3147 independent ones (R_{int} = 0.0322). Monoclinic crystals, a=9.9203(3), b=8.7798(3), c=15.9274(5) Å, β=98.757(1)°, V=1371.08(8) Å3, Z=4 ($C_{14}H_{16}N_2O_4$), spatial group $P_{21/n}$, d=1.338 g/cm3, μ = 0.099 mm^{-1}. The initial array of measured intensities was processed and absorption was taken into account using the SAINT [36] and SADABS [37] program (multi-scan, T_{min}=0.931, T_{max}=0.983) [37].

The structure is deciphered by the direct method. The positions of nonhydrogen atoms are refined in an anisotropic approximation by full-matrix the least squares method. Hydrogen atoms were placed in geometrically calculated positions and their positions were refined in an isotropic approximation with fixed positional and thermal parameters (the "rider" model). The calculations used 2562 reflections of independent reflections with $I \geq 2\sigma(I)$, the number of specified parameters is 182. The final divergence factors are: R_1 0.0413, wR_2 0.1137 (for reflections with $I \geq 2\sigma(I)$), R_1 0.0525, wR_2 0.1247 (for all reflections), GooF=1.059. Residual density peaks: Δρ = 0.227 and -0.161 e/Å3. The structure was deciphered and refined according to the programs "SHELXT 2014/5" [38] and "SHELXL-2018/3" [39]. The RSA data in the form of a CIF file is deposited at the Cambridge Crystal Structure Data Center (CCDC 2358257).

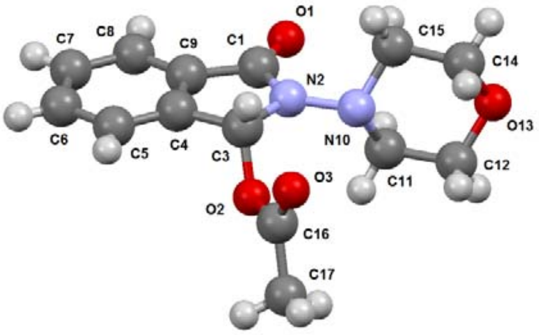

Table S1. Atomic coordinates (x 10^4) and equivalent isotropic displacement parameters (E^2x 10^3) for compound **15**. U(eq) is defined as one third of the trace of the orthogonalized U^{ij} tensor.

Compound **15**	x	y	z	U(eq)
O(1)	3914(1)	7854(1)	4939(1)	53(1)
O(2)	5219(1)	7516(1)	2405(1)	43(1)
O(3)	6681(2)	5720(2)	2111(1)	84(1)
C(1)	3887(1)	7409(1)	4214(1)	39(1)
N(2)	4996(1)	7035(1)	3835(1)	42(1)
C(3)	4658(1)	6503(2)	2968(1)	40(1)
C(4)	3125(1)	6608(2)	2823(1)	43(1)
C(5)	2209(2)	6301(2)	2101(1)	60(1)
C(6)	830(2)	6539(2)	2142(1)	70(1)
C(7)	394(2)	7031(2)	2878(1)	68(1)
C(8)	1316(2)	7335(2)	3601(1)	56(1)
C(9)	2689(1)	7123(2)	3557(1)	42(1)
N(10)	6381(1)	7197(1)	4182(1)	40(1)
C(11)	6773(2)	6255(2)	4934(1)	54(1)
C(12)	8293(2)	6439(2)	5212(1)	69(1)

O(13)	8672(1)	7968(1)	5380(1)	63(1)
C(14)	8260(2)	8880(2)	4651(1)	54(1)
C(15)	6746(1)	8793(2)	4362(1)	46(1)
C(16)	6250(2)	6984(2)	2020(1)	48(1)
C(17)	6753(2)	8171(2)	1485(1)	62(1)

Table S2. Bond lengths [E] and angles [°] for compound **15**.

Bond	e	Bond	e
O(1)-C(1)	1.2158(16)	C(1)-N(2)-C(3)	114.25(11)
O(2)-C(16)	1.3525(16)	N(10)-N(2)-C(3)	118.58(10)
O(2)-C(3)	1.4350(16)	O(2)-C(3)-N(2)	109.91(10)
O(3)-C(16)	1.191(2)	O(2)-C(3)-C(4)	110.22(11)
C(1)-N(2)	1.3731(16)	N(2)-C(3)-C(4)	102.07(10)
C(1)-C(9)	1.4804(19)	C(5)-C(4)-C(9)	121.12(14)
N(2)-N(10)	1.4076(15)	C(5)-C(4)-C(3)	129.65(13)
N(2)-C(3)	1.4479(16)	C(9)-C(4)-C(3)	109.22(11)
C(3)-C(4)	1.5051(18)	C(4)-C(5)-C(6)	117.27(16)
C(4)-C(5)	1.380(2)	C(7)-C(6)-C(5)	121.56(16)
C(4)-C(9)	1.3823(19)	C(6)-C(7)-C(8)	121.00(16)
C(5)-C(6)	1.394(3)	C(7)-C(8)-C(9)	117.49(16)
C(6)-C(7)	1.379(3)	C(4)-C(9)-C(8)	121.55(14)
C(7)-C(8)	1.383(2)	C(4)-C(9)-C(1)	109.39(11)
C(8)-C(9)	1.387(2)	C(8)-C(9)-C(1)	129.05(13)
N(10)-C(11)	1.4587(18)	N(2)-N(10)-C(11)	112.80(11)
N(10)-C(15)	1.4646(17)	N(2)-N(10)-C(15)	111.76(10)
C(11)-C(12)	1.514(2)	C(11)-N(10)-C(15)	110.79(11)
C(12)-O(13)	1.409(2)	N(10)-C(11)-C(12)	107.96(13)
O(13)-C(14)	1.4191(19)	O(13)-C(12)-C(11)	112.49(13)
C(14)-C(15)	1.504(2)	C(12)-O(13)-C(14)	110.27(12)
C(16)-C(17)	1.480(2)	O(13)-C(14)-C(15)	111.84(12)
C(16)-O(2)-C(3)	117.49(11)	N(10)-C(15)-C(14)	108.29(11)
O(1)-C(1)-N(2)	126.20(13)	O(3)-C(16)-O(2)	123.34(14)
O(1)-C(1)-C(9)	128.72(12)	O(3)-C(16)-C(17)	125.42(14)
N(2)-C(1)-C(9)	105.06(11)	O(2)-C(16)-C(17)	111.25(13)
C(1)-N(2)-N(10)	127.07(11)		

Table S3. Torsion angles [°] for compound **15**.

Angle	ω
O(1)-C(1)-N(2)-N(10)	-5.2(2)
C(9)-C(1)-N(2)-N(10)	175.9(1)
O(1)-C(1)-N(2)-C(3)	178.6(1)
C(9)-C(1)-N(2)-C(3)	-0.3(2)
C(16)-O(2)-C(3)-N(2)	111.4(1)
C(16)-O(2)-C(3)-C(4)	-136.9(1)
C(1)-N(2)-C(3)-O(2)	117.8(1)
N(10)-N(2)-C(3)-O(2)	-58.7(2)
C(1)-N(2)-C(3)-C(4)	0.9(2)
N(10)-N(2)-C(3)-C(4)	-175.7(1)
O(2)-C(3)-C(4)-C(5)	60.9(2)
N(2)-C(3)-C(4)-C(5)	177.6(2)
O(2)-C(3)-C(4)-C(9)	-117.8(1)
N(2)-C(3)-C(4)-C(9)	-1.1(1)
C(9)-C(4)-C(5)-C(6)	0.2(2)
C(3)-C(4)-C(5)-C(6)	-178.3(2)
C(4)-C(5)-C(6)-C(7)	-1.2(3)
C(5)-C(6)-C(7)-C(8)	1.2(3)
C(6)-C(7)-C(8)-C(9)	-0.1(3)
C(5)-C(4)-C(9)-C(8)	0.8(2)
C(3)-C(4)-C(9)-C(8)	179.6(1)
C(5)-C(4)-C(9)-C(1)	-177.9(1)
C(3)-C(4)-C(9)-C(1)	1.0(2)
C(7)-C(8)-C(9)-C(4)	-0.9(2)
C(7)-C(8)-C(9)-C(1)	177.5(2)
O(1)-C(1)-C(9)-C(4)	-179.3(1)
N(2)-C(1)-C(9)-C(4)	-0.4(2)
O(1)-C(1)-C(9)-C(8)	2.2(2)
N(2)-C(1)-C(9)-C(8)	-179.0(1)
C(1)-N(2)-N(10)-C(11)	63.6(2)
C(3)-N(2)-N(10)-C(11)	-120.3(1)
C(1)-N(2)-N(10)-C(15)	-62.0(2)
C(3)-N(2)-N(10)-C(15)	114.1(1)
N(2)-N(10)-C(11)-C(12)	176.1(1)
C(15)-N(10)-C(11)-C(12)	-57.8(2)

N(10)-C(11)-C(12)-O(13)	57.6(2)
C(11)-C(12)-O(13)-C(14)	-57.8(2)
C(12)-O(13)-C(14)-C(15)	58.0(2)
N(2)-N(10)-C(15)-C(14)	-174.7(1)
C(11)-N(10)-C(15)-C(14)	58.6(2)
O(13)-C(14)-C(15)-N(10)	-58.2(2)
C(3)-O(2)-C(16)-O(3)	1.9(2)
C(3)-O(2)-C(16)-C(17)	-178.0(1)

EuroVector Elemental Analyser

1) **N-(2-bromo-3-phenylallidene)morpholine-4-amine (8).**

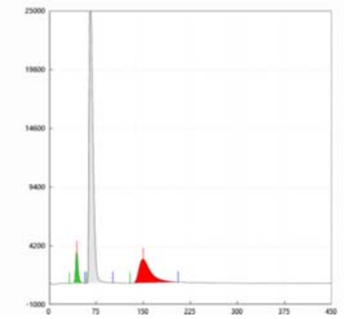

Element	Element %	Area(µV*sec)	Ret.Time (sec)	Start (sec)	End (sec)
Nitrogen.	10.220	125,570.7	439	327	572
Carbon	51.298	1,831,578.8	657	595	1,015
Hydrogen	4.921	459,317.6	1,495	1,293	2,050
Sulphur	-	-	-	-	-
Oxygen	-	-	-	-	-

2) **N-(4-(styryl)benzylidene)morpholine-4-amine (9).**

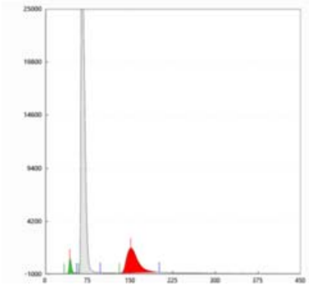

Element	Element %	Area(µV*sec)	Ret.Time (sec)	Start (sec)	End (sec)
Nitrogen.	5.736	66,862.6	437	331	553
Carbon	78.051	2,469,730.8	649	591	972
Hydrogen	6.627	564,473.6	1,509	1,304	2,009
Sulphur	-	-	-	-	-
Oxygen	-	-	-	-	-

3) **4-bromo-2-((morpholinoamino)methyl)phenol (10).**

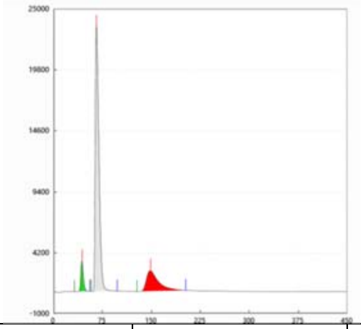

Element	Element %	Area(µV*sec)	Ret.Time (sec)	Start (sec)	End (sec)
Nitrogen.	10.326	117,435.4	438	323	560
Carbon	45.056	1,480,112.5	660	580	981
Hydrogen	4.410	369,397.5	1,485	1,283	2,025
Sulphur	-	-	-	-	-
Oxygen	-	-	-	-	-

4) **N-(pyridine-4-ilmethyl)morpholine-4-amine (11).**

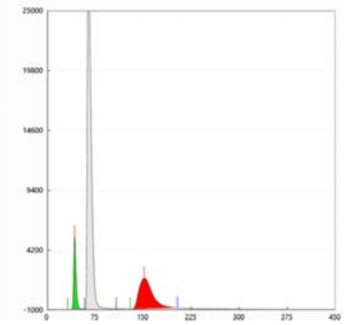

Element	Element %	Area(µV*sec)	Ret.Time (sec)	Start (sec)	End (sec)
Nitrogen.	21.127	277,061.5	432	320	586
Carbon	61.318	2,184,701.2	646	586	1,082
Hydrogen	6.609	638,651.9	1,521	1,297	2,035
Sulphur	-	-	-	-	-
Oxygen	-	-	-	-	-

5) **2-ethoxy-4-((morpholinoamino)methyl)-phenol (12).**

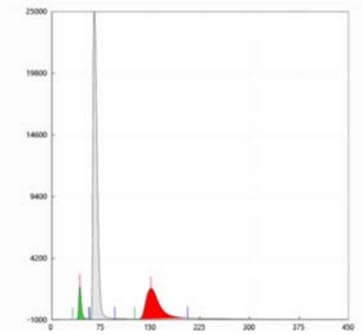

Element	Element %	Area(µV*sec)	Ret.Time (sec)	Start (sec)	End (sec)

Nitrogen.	10.923	125,210.8	432	326	565
Carbon	61.269	1,899,993.2	650	589	963
Hydrogen	7.099	597,308.5	1,510	1,264	2,064
Sulphur	-	-	-	-	-
Oxygen	-	-	-	-	-

6) 2-((Morpholinoamino)methyl)benzoic acid (13).

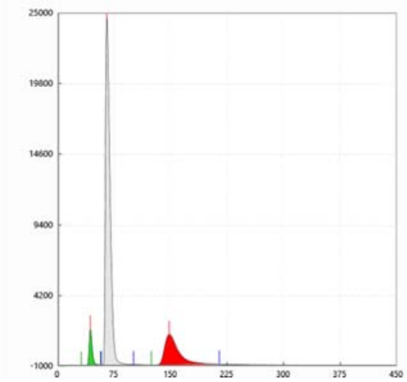

Element	Element %	Area(µV*sec)	Ret.Time (sec)	Start (sec)	End (sec)
Nitrogen.	11.436	122,346.3	433	318	580
Carbon	61.419	1,774,511.9	654	573	1,006
Hydrogen	6.133	476,404.4	1,483	1,242	2,145
Sulphur	-	-	-	-	-
Oxygen	-	-	-	-	-
